# HSV-1 Glycoprotein D and Its Surface Receptors: Evaluation of Protein–Protein Interaction and Targeting by Triazole-Based Compounds through In Silico Approaches

**DOI:** 10.3390/ijms24087092

**Published:** 2023-04-11

**Authors:** Roberta Bivacqua, Isabella Romeo, Marilia Barreca, Paola Barraja, Stefano Alcaro, Alessandra Montalbano

**Affiliations:** 1 Dipartimento di Scienze e Tecnologie Biologiche Chimiche e Farmaceutiche (STEBICEF), Università degli Studi di Palermo, Via Archirafi 32, 90123 Palermo, Italy; 2 Dipartimento di Scienze della Salute, Università degli Studi “Magna Græcia” di Catanzaro, Campus “S. Venuta”, Viale Europa, 88100 Catanzaro, Italy; 3 Net4Science Academic Spin-Off, Università degli Studi “Magna Græcia” di Catanzaro, Campus “S. Venuta”, Viale Europa, 88100 Catanzaro, Italy

**Keywords:** protein–protein interaction, HSV-1, 1,2,3-triazoles, docking, molecular dynamics simulations, glycoprotein D

## Abstract

Protein–protein interactions (PPI) represent attractive targets for drug design. Thus, aiming at a deeper insight into the HSV-1 envelope glycoprotein D (gD), protein–protein docking and dynamic simulations of gD-HVEM and gD-Nectin-1 complexes were performed. The most stable complexes and the pivotal key residues useful for gD to anchor human receptors were identified and used as starting points for a structure-based virtual screening on a library of both synthetic and designed 1,2,3-triazole-based compounds. Their binding properties versus gD interface with HVEM and Nectin-1 along with their structure-activity relationships (SARs) were evaluated. Four [1,2,3]triazolo[4,5-*b*]pyridines were identified as potential HSV-1 gD inhibitors, for their good theoretical affinity towards all conformations of HSV-1 gD. Overall, this study suggests promising basis for the design of new antiviral agents targeting gD as a valuable strategy to prevent viral attachment and penetration into the host cell.

## 1. Introduction

Herpesviridae is a large family of enveloped double-stranded DNA viruses that includes eight human herpesviruses (HHV) divided into three subfamilies: alphaherpesvirinae (herpes simplex virus [HSV] and varicella-zoster virus [VZV]); betaherpesvirinae (cytomegalovirus [CMV], HHV-6, and HHV-7); and gammaherpesvirinae (Epstein–Barr virus [EBV] and HHV-8) [1]. All herpesviruses, especially those belonging to the alphaherpesvirinae subfamily, are able to establish latent infections that can be reactivated by endogenous or exogenous stimuli, causing clinical symptoms [2]. HSV infections are widely spread around the world, thus representing a considerable public health issue. There are two main types of HSV that infect humans, HSV-1 and HSV-2. Both are responsible for genital herpes, although HSV-1 mostly causes oral infections and is acquired during childhood [3]. HSV-1 infection can also lead to complications, such as keratitis, encephalitis, meningitis, and systemic disease in neonates and immune-compromised patients [4,5]. Currently, antiviral drugs available for the treatment of herpesvirus infections include acyclovir (ACV), penciclovir, valacyclovir, famciclovir, foscarnet, and cidofovir, which terminate viral DNA synthesis by inhibiting the viral DNA polymerase [6]. However, these drugs are not effective in the complete eradication of the infection, but only in reducing the frequency and duration of the episodes. In addition, both the emergence of drug-resistant HSV-1 strains, in particular for immune-compromised patients, and drug toxicity issues, increase the need for new antiviral agents acting with different mechanisms of action from those currently in use [1,7].

An interesting strategy for the treatment of viral infections is to prevent viral attachment and entry into the host cell, a complex process mediated by the interaction of different viral envelope glycoproteins with specific host surface receptors [8]. The HSV-1 envelope contains 11 glycoproteins involved in the early stages of viral attachment and penetration [2]. Among them, gB, gC, gD, gH, and gL are considered essential for cell entry. Attachment to the host membrane begins with the interaction of gB and gC with cell surface heparan sulfate (HS) proteoglycans, which brings the virus closer to the cell [9,10]. The binding of gD to one of the specific cellular receptors and the consequent conformational change that occurs in the structure of gD trigger a cascade of events that promote the formation of the core fusion machinery with gB and the gH/gL complex that allows virus entry [11]. HSV-1 gD can bind three classes of receptors, depending on the cell type:

1. Herpesvirus entry mediator (HVEM), a member of the tumor necrosis factor receptor superfamily (TNFR) expressed on activate lymphocytes and in other human tissues including kidney, lung, and liver;
2. Nectin-1 and 2, immunoglobulin (Ig)-like cell adhesion molecules (CAMs) expressed on the surface of neuronal and epithelial cells [12];
3. 3-O-sulfated heparan sulfate (3-OS HS), whose biological role has not yet been well clarified [13].

Due to the crucial role that gD plays in host cell fusion, a thorough knowledge of its structural features and of the molecular mechanisms regulating its activity may be useful to develop new inhibitors. 

The current study aims at a deeper insight into gD and its cellular receptor interactions by means of computational methods, taking advantage of the HSV-1 gD experimentally solved structures in complex to HVEM and Nectin-1. The complexes resulting from protein–protein docking experiments were submitted to molecular dynamic simulations (MDs). GBPM and free energy calculation analyses revealed the most stable complexes and their pivotal points of interaction. Considering that protein–protein interactions (PPI) represent attractive targets for drug design, a structure-based virtual screening (SBVS) was performed taking into account the key residues useful for gD to anchor human receptors. 

**Figure 1:**
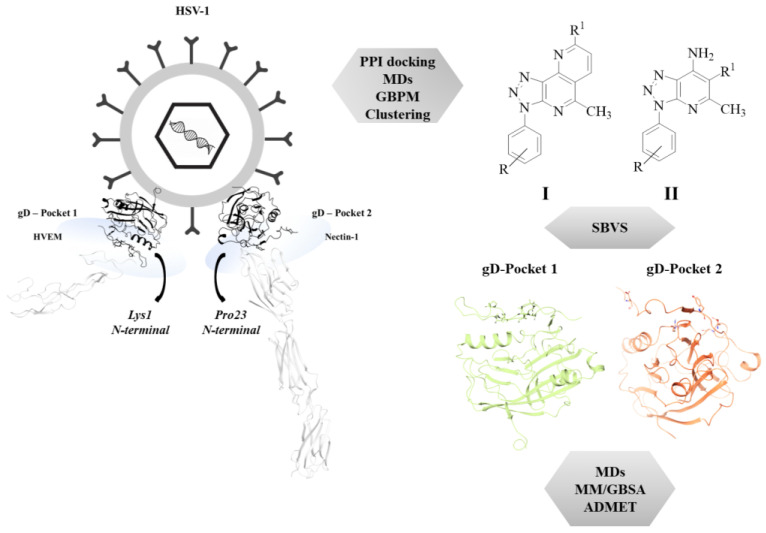
Graphical workflow of the applied in silico approaches on HSV-1 gD Pockets.

Much effort has been devoted by our research group in the identification of small molecules as bioactive compounds [14,15,16,17] and among them, we have recently reported a small set of [1,2,3]triazolo[4,5-*h*][1,6]naphthyridines as promising photosensitizer agents [18]. Both 1,2,3-triazole based compounds [19,20,21,22] and naphthyridine derivatives have been reported in the literature for their promising antiviral activity, refs. [23,24,25,26,27] more specifically, 1,2,3 triazole ring and 1,6-naphthyridine core appear as recurring moieties in heterocyclic compounds exhibiting anti-HSV-1 properties at the micromolar level [28,29,30,31,32]. Hence, we used a small set of 12 synthetic [1,2,3]triazolo[4,5-*h*][1,6]naphthyridines (I, Figure 1 and **1–12**, Table 1) to evaluate their ability to target gD binding interface with HVEM and Nectin-1. Moreover, 63 novel [1,2,3]triazolo[4,5-*h*][1,6]naphthyridines were designed (**19–81**, Table 1) in order to evaluate their binding properties into gD pockets and to explore their structure-activity relationships (SARs). Furthermore, since triazolopyridine systems have been extensively studied as interesting scaffolds exhibiting antiviral activities, [33,34,35] we also investigated the binding affinity of [1,2,3]triazolo[4,5-*b*]pyridines (II, Figure 1 and **13–18 Table 1**), synthetic precursors of the aforementioned triazolo-naphthyridines, versus gD interface with HVEM and Nectin-1. The workflow is reported in Figure 1.

## 2. Results and Discussion

In order to investigate the binding affinity of gD for its surface receptors and to gain useful structural insights regarding the pivotal interactions occurring at the gD interface, we performed a knowledge-based protein–protein docking employing HADDOCK 2.4 web-server [36]. Due to the involvement of gD both in cell adhesion function and viral entry mechanism depending on its cellular receptors, we considered the X-ray structure of gD both in complex to HVEM (PDB code: 1JMA) and Nectin-1 (PDB code: 3U82), respectively at 2.65 and 3.16 Å resolution. The complexes were prepared using Protein Preparation Wizard and uploaded in PDB format to the HADDOCK server. The input docking parameters were discussed in the Section 3.1. All the generated complexes were clustered according to their HADDOCK score, calculated as a weighted sum of a variety of energy terms (including van der Waals, electrostatic, desolvation, and restraint violation energies) and buried surface area (BSA); the Z-score value was also calculated to select the best cluster with respect to all obtained clusters: the most negative Z-score is indicative of the top ranked cluster. For the gD-HVEM and gD-Nectin-1 complexes, we obtained 188 structures gathered in 9 clusters (Figure S1), and 198 structures in 2 clusters (Figure S2), respectively. For both complexes, the docking results of each cluster were reported in Table 2.

**Figure 2:**
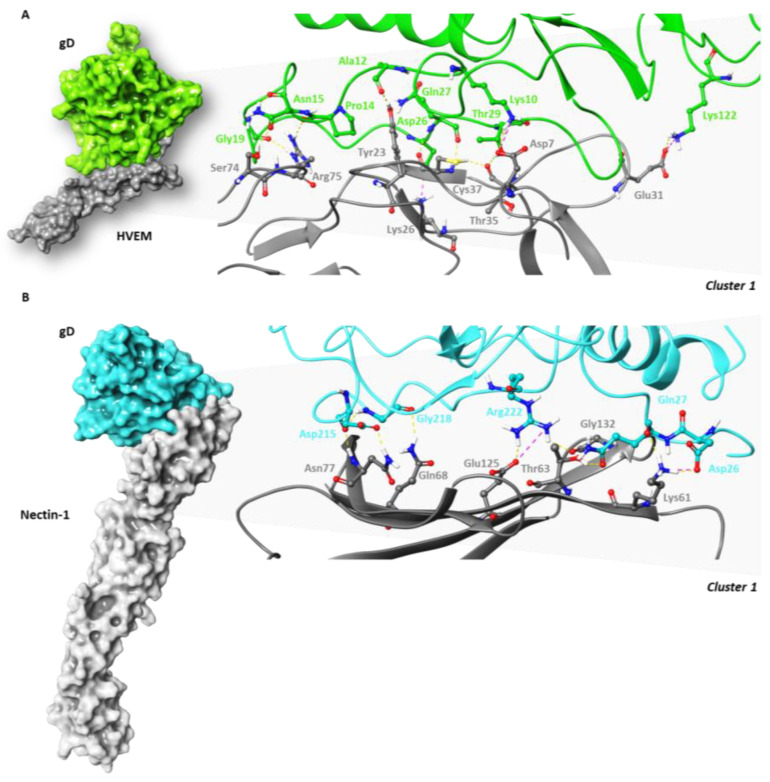
Surface representation of (**A**) gD-HVEM and (**B**) gD-Nectin-1 complexes and focus on the interface with key interactions labelled and displayed in carbon sticks.

The best clusters (the lowest in energy) were analysed in terms of interactions using Maestro graphical user interface (Figure 2) and were aligned to the experimental structures through the Protein Structure Alignment tool.

By overlapping (Figure S3), it resulted that for gD-HVEM and gD-Nectin-1 complexes, the RMSD value between the best docked pose and the X-ray structure was 1.54 and 0.80 Å, respectively. All the residues involved in the PPIs are summarized in Table 3.

**Figure 3:**
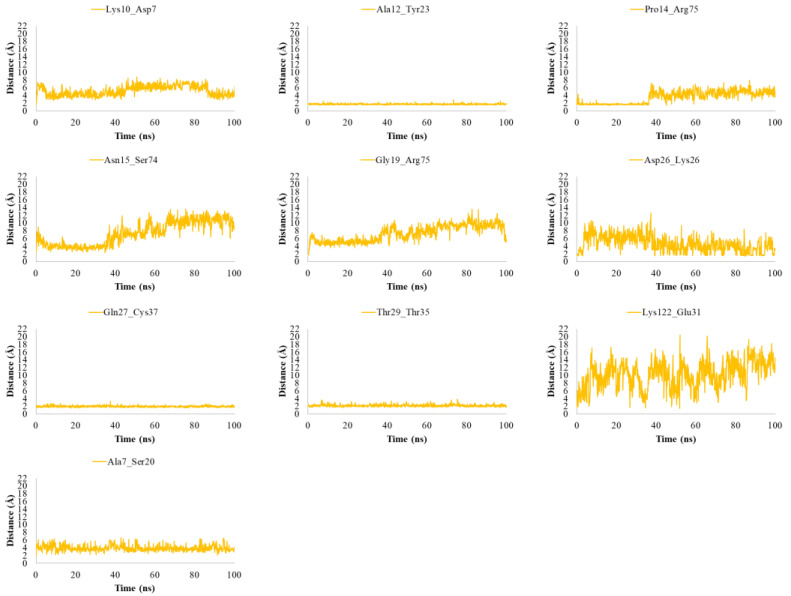
Plots of the distances between the residues of gD involved in the interaction at the interface with HVEM after 100 ns of MDs.

The best obtained docked structures for each complex were refined using Protein Preparation Wizard and energy minimized with OPLS_2005 force field [37]. In order to explore any potential conformational changes of the gD interface, we submitted 100 ns of MDs for both complexes. The stability of MDs trajectories was monitored by the RMSD trend of the protein’s backbone atoms from its initial conformation. The average RMSD values of gD-HVEM and gD-Nectin-1 complexes were 3.05 Å and 2.50 Å, respectively. By monitoring the distances between the interface’s residues for gD-HVEM, we observed that several interactions previously detailed in Table 3 were maintained during MDs run, except for Asn15, Gly19, and Lys122 of gD, which were subjected to greater fluctuations, thus preventing the contacts with HVEM residues. Instead, Ala7 gained contacts with Ser20 during the whole MDs (Figure 3).

**Figure 4:**
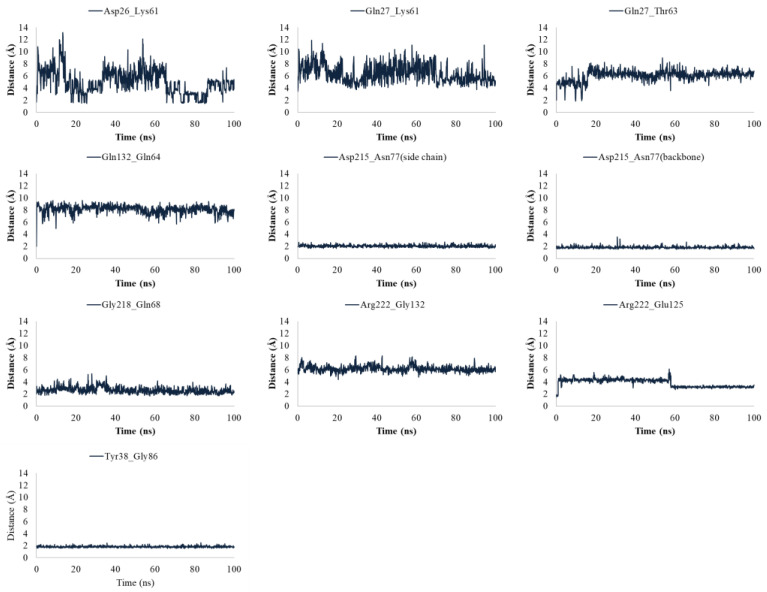
Plots of the distances between the residues of gD involved in the interaction at the interface with Nectin-1 after 100 ns of MDs.

Regarding gD in complex to Nectin-1, the interactions engaged by Gln27 and Gln132 of gD at the interface with Nectin-1 were lost, but it was found that Tyr38 was located in proximity to Gly86 of Nectin-1 during MDs (Figure 4).

To better characterize the gD interface, a deeper analysis using GBPM was performed on all frames of MDs for each system. This method helps to map the key hotspots responsible for PPI by combining GRID molecular interaction fields (MIFs) according to the GRAB tool algorithm [38]. We considered gD as guest and HVEM and Nectin-1 as hosts. Three GRID probes, such as DRY, N1, and O, were chosen to mimic the hydrophobic, H-bond donor, and acceptor areas, respectively. Taking into account an energy threshold above the 30% from the global energy minimum GRID points, we summarized the pivotal residues up to 3 Å from GBPM points. The contribution of each residue was derived by the summa of its GBPM points energy in the matching frames (see Tables S1 and S2). After calculating the average score based on the total number of frames, the key hotspots were split into quartiles to increase the clarity for the reader: quartile 1 (Q1) includes the residues with the major contribution to PPI until quartile 4 (Q4), which contains residues with the weakest interactions, during the entire trajectories (Table 4).

**Figure 5:**
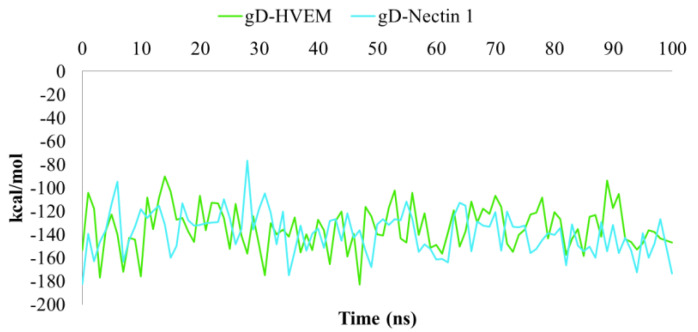
Plot of MM/GBSA trend for HVEM and Nectin-1 in complex to gD during 100 ns of MDs.

For both protein–protein complexes, 100 frames extracted by MDs were adopted to calculate the relative binding free energy (ΔGbind) using Molecular Mechanics/Generalized Born Surface Area (MM/GBSA) methodology, as applied in a recent study [39]. The results of the calculated ΔGbind trend for gD-HVEM and gD-Nectin-1 are depicted in Figure 5. Even showing a similar energy profile, the average values of −134.36 and −138.26 kcal/mol were turned out for HVEM and Nectin-1 in complex to gD, respectively, thus resulting in a more stabilizing effect of gD towards Nectin-1.

After investigating the PPIs’ structural details, we focused on the key residues of gD liable for the interaction with the analyzed human receptors to find potential ligands able to prevent the connection with them. According to the literature [40], several gD N-terminus residues at position 7–15 and 26–29 are responsible for the bind with HVEM, whereas two residues, such as Asp26 and Tyr38, also belonging to gD N-terminus, are relevant for the connection with Nectin-1 [41]. Apart from the shared Asp26 and Gln27 residues, both human receptors interact with different portions of gD. It was underlined that HVEM and Nectin-1 show non-reciprocal competition for binding to gD [42]. Indeed, the interaction between HVEM and gD induces a conformational change in gD that results in the formation of the N-terminal hairpin structure that masks the binding site of Nectin-1 [42]. Similarly, the interaction with Nectin-1 induces a new conformation of the gD N-terminus that prevents the HVEM binding [4]. Considering therefore that gD is not able to bind both receptors at the same time [41], we evaluated the possible conformational changes of gD during MDs. As previously reported [43], MDs trajectories were clustered based on RMSD matrix using the average hierarchical clustering linkage method, obtaining three representative structures of gD. Taking into account that the pivotal interactions of gD with HVEM and Nectin-1 involve distinct residues, we used all three representative structures generated from each complex (Figure S4) as starting point for SBVS.

Thus, we performed molecular docking simulations of an in-house small library of 12 [1,2,3] triazolo [4,5-*h*] [1,6] naphthyridines (**1–12**, Table 1) with their precursors [1,2,3] triazolo [4,5-*b*] pyridines (**13–18**, Table 1) on each binding site, separately, using the docking program Glide in SP mode. Additionally, with the aim of exploring the influence of electron-withdrawing and/or electron-donating substituents at N-3 and C-8 positions of the triazolo-naphthyridine core on gD-ligand interaction, a focused library of 63 triazolo [4,5-*h*] [1,6] naphthyridines (**19–81**, Table 1) was also designed, along with the corresponding [1,2,3]triazolo [4,5-*b*] pyridines (**82–85**, Table 1).

We calculated the average G-score value for each cluster, aiming to focus on the compounds able to better recognize both Pocket 1 and 2 (Table S3). From the docking results, it clearly emerged that the tricyclic [1,2,3] triazolo [4,5-*h*] [1,6] naphthyridine moiety showed a lower ability to recognize both the binding pockets compared to the bicyclic triazolo [4,5-*b*] pyridine derivatives. Furthermore, regardless of the electronic nature of the substituents at the N-3 phenyl ring of the triazolo-naphthyridine core, no significant differences could be observed. 

Concerning triazolo [4,5-*b*] pyridine derivatives, the presence of the 3,4,5-trimethoxylphenyl ring at N-3 reduced the theoretical binding affinity to gD. Conversely, better results were obtained with *p*-phenyl substituted derivatives bearing the hydroxymethyl group at C-6 (**14**, **16**, **83**, **85**).

**Figure 6:**
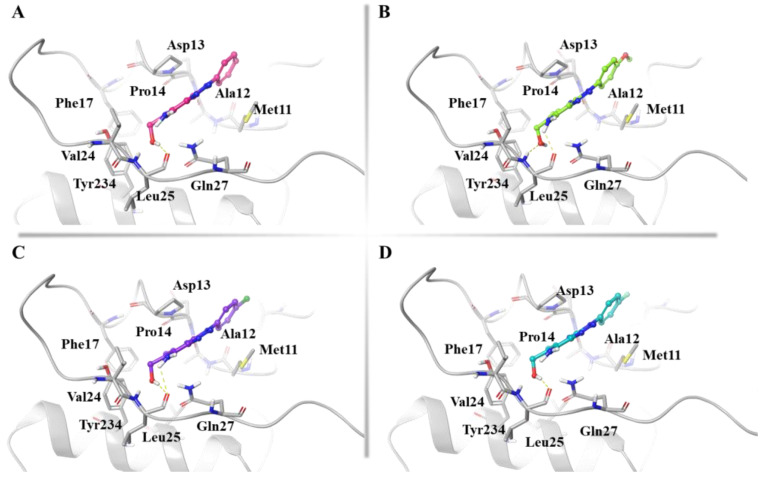
3D representation of (**A**) **14**, (**B**) **16**, (**C**) **83**, and (**D**) **85** in complex to the most populated cluster of gD-Pocket 1. gD-Pocket 1 is illustrated in gray, with the residues involved in pivotal contacts shown as carbon sticks. **14**, **16**, **83**, and **85** are depicted as pink, green, violet, and cyan carbon sticks, and H-bonds are indicated as yellow dashed lines.

Overall, among the 85 investigated compounds, we focused on the binding mode of the compounds able to recognize all the representative structures of both pockets with average G-score values lower than −5.00 kcal/mol. Accordingly, four triazole-pyridines derivatives, **14** (Figure S5), **16** (Figure S6), **83** (Figure S7), and **85** (Figure S8) showed a favored energetic profile in complex with all gD conformations. In detail, considering the most populated cluster resulted for gD-Pocket 1, the hydroxyl group of **14** (Figure 6A), **16** (Figure 6B), **83** (Figure 6C), and **85** (Figure 6D) was anchored to Leu25. Moreover, **16** and **83** formed an additional H-bond between the amino group and Leu25.

**Figure 7:**
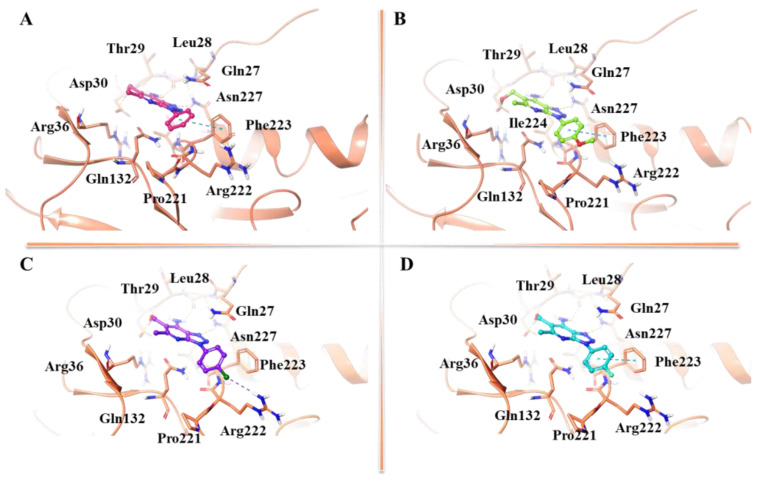
3D representation of (**A**) **14**, (**B**) **16**, (**C**) **83**, and (**D**) **85** in complex to the most populated cluster of gD-Pocket 2. gD-Pocket 2 is illustrated in salmon, with the residues involved in pivotal contacts shown as carbon sticks. Compounds **14**, **16**, **83**, and **85** are depicted as pink, green, violet, and cyan carbon sticks, whereas H-bonds and π-π interactions are indicated as yellow and cyan dashed lines, respectively.

In gD-Pocket 2, **14**, **16**, **83**, and **85** engaged several H-bonds with Leu28, Asp30, Asn227, and a π-π stacking interaction with Phe223, probably due to the absence of the N-terminal extension (Figure 7).

**Figure 8:**
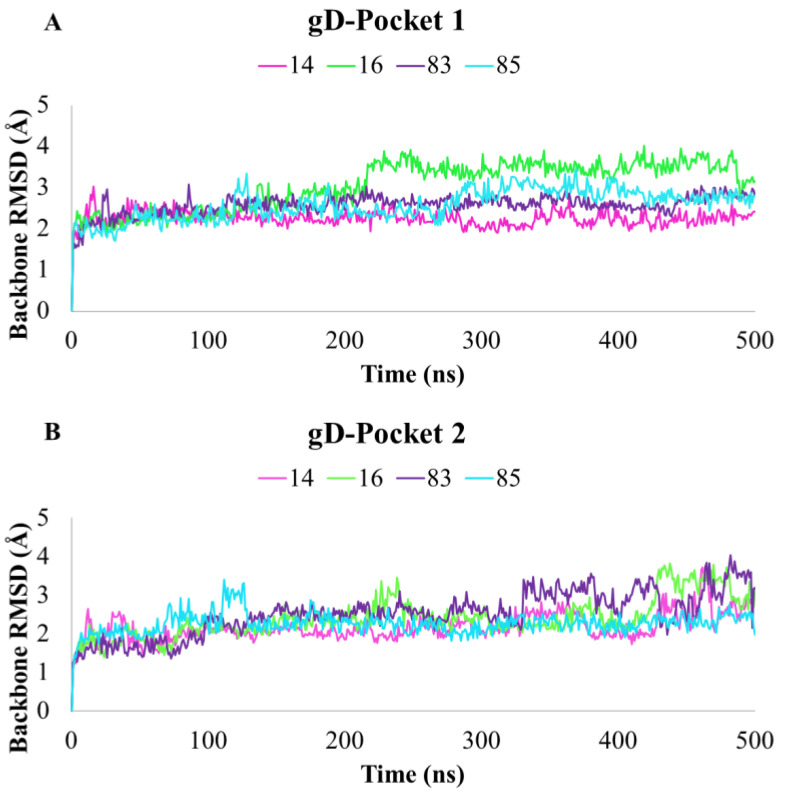
RMSD plots of **14**, **16**, **83**, and **85** compounds in complex with (**A**) gD-Pocket 1 and (**B**) gD-Pocket 2, calculated on protein’s backbone atoms during 500 ns of MDs.

The best docked poses of **14**, **16**, **83,** and **85** in complex with the most representative cluster of gD-Pocket 1 and gD-Pocket 2 were submitted to 500 ns of MDs using Desmond [44]. The results of MDs were investigated in terms of stability and conformational flexibility in the presence of the selected compounds. The stability of the complexes was evaluated by calculating the RMSD of the protein’s backbone atoms from its initial to final conformation over the whole simulation. By RMSD analysis, we observed that the most promising compounds maintained overall stability throughout MDs in both Pockets, as shown in Figure 8. In particular, for Pocket 1, the average RMSD values of 2.27, 3.09, 2.58, and 2.59 Å (Figure 8A) were computed for **14**, **16**, **83**, and **85**, respectively. On the other hand, for Pocket 2, we observed average RMSD values in the range of 2.17–2.51 Å (Figure 8B).

Furthermore, for each system, the binding free energy ΔGbind was calculated using MM/GBSA methodology, extracting 100 snapshots from 500 ns of MDs. MM/GBSA analysis showed that the average calculated ΔGbind of **14**, **16**, **83**, and **85** complexed with gD-Pocket 1 were −38.88, −36.52, −40.22, and −31.14 kcal/mol, respectively, during the entire trajectories. The average ΔGbind of **14**, **16**, **83**, and **85** in complex to gD-Pocket 2 were −31.78, −29.71, −36.28, and −34.63 kcal/mol, respectively. ADME parameters of the most promising compounds were predicted using the SwissADME server, and the obtained data are reported in Table 5. 

All the investigated compounds are achiral, fit Lipinski’s rule of five, and were not found to be pan assay interference compounds (PAINS), thus resulting suitable for further HSV-1 in vitro investigation.

## 3. Materials and Methods

### 3.1. Computational Methods

#### 3.1.1. Protein–protein Preparation of gD-HVEM and gD-Nectin-1 and Docking Simulations

All computational studies were carried out using Schrödinger Suite 2018-1 [45]. The X-ray crystallographic structures of gD in complex with HVEM (PDB code: 1JMA) [46] and with Nectin-1 (PDB code: 3U82) [41] were used. Both gD structures were prepared and optimized using the Maestro Protein Preparation Wizard tool [47] with OPLS_2005 [37] as force field at pH 7.4. The structures were also optimized by the addition of missing loops using Prime software [44,48] and the determination of the protonation state of the ionizable amino acid residues by means of the Epik program [49]. Knowledge-based protein–protein docking of gD with HVEM and Nectin-1 was performed with HADDOCK 2.4 (High Ambiguity-Driven biomolecular DOCKing) web-server [36]. For each complex, the sampling parameters were as follows: 1000 structures for rigid-body docking, 200 structures for the final refinement, and a cut-off equal to 5.0 to define neighboring flexible regions. For the complex gD-HVEM from 1JMA model, gD amino acids Ala7, Ser8, Leu9, Lys10, Met11, Ala12, Asp13, Pro14, Asn15, Val24, Leu25, Asp26, Gln27, Leu28, Thr29, Asp30, Pro31, Pro32 and HVEM amino acids Pro17, Lys18, Cys19, Ser20, Pro21, Gly22, Tyr23, Arg24, Val25, Lys26, Gly30, Glu31, Leu32 Thr33, Gly34, Thr35, Val36, Cys37, Glu38, Pro39, Ser74, Arg75, and Thr76 were considered as active residues, whereas passive residues were automatically identified as residues surrounding the active ones before submitting the docking job. For the complex gD-Nectin-1 from 3U82 model, gD amino acids Pro23, Leu25, Gln27, Arg36, Val37, Tyr38, His39, Gln132, Val214, Asp215, Ser216, Ile217, Gly218, Met219, Leu220, Pro221, Arg222, Phe223, Thr230, Val231, Tyr234 and Nectin-1 amino acids Ser59, Lys61, Thr63, Gln64, Thr66, Gln68, Lys75, Gln76, Asn77, Ile80, Tyr81, Asn82, Met85, Gly86, Val87, Ser88, Leu90, Glu125, Ala127, Thr128, Phe129, Pro130, Thr131, Gly132, and Asn133 were considered as active residues, whereas passive residues were again automatically identified. For each complex, the docked structures were loaded on Maestro interface of the Schrödinger software in PDB format for visual inspection and for the following in silico analysis.

#### 3.1.2. MDs, GBPM, and MM/GBSA Calculations of gD-HVEM and gD-Nectin-1 Complexes

The best docked poses of each complex were submitted to 100 ns of MDs using Desmond ver. 4.2 [44]. To perform simulations in an aqueous biological environment, an appropriate system was built using OPLS_2005 as force field and an orthorhombic box with TIP4P water model extending of 10 Å outside the complex in all sides. The systems were maintained at a salt concentration of 0.15 M by adding appropriate Cl^−^ counter ions to neutralize them to maintain the physiological condition. After optimization of the solvated models, we relaxed the systems with the Martyna−Tobias−Klein isobaric−isothermal ensemble (MTK_NPT). Finally, 100 ns unconstrained MDs were carried out using the following conditions: the NPT ensemble, a constant temperature of 300 K, a pressure of 1 bar, and a recording interval equal to 100 ps both for energy and for trajectory collecting 1000 frames for each simulation. 

For both complexes, all frames were considered for GBPM analysis [38]. As previously reported [50], in order to evidence hydrophobic and hydrogen bond donors and acceptors spots, we used DRY, N1, and O GRID probes, respectively. For each complex, gD was seen as guest and HVEM and Nectin-1 as hosts. The selected residues at the interface covered a maximum distance of 3 Å from the most relevant interaction energy points (GBPM features) of the computed molecular interaction fields (MIFs). After selecting an energy cutoff 30% above the global minimum, the pivotal hotpots were resulted by the summa of the related GBPM features interaction energy. 

One thousand snapshots from 100 ns of MDs were applied for the Molecular Mechanics/Generalized Born Surface Area (MM/GBSA) free energy calculations [51] based on the following equation:

ΔG_bind_ = G_comp_ − G_pro_ −G_lig_ = ΔE_ele_ + ΔE_vdw_ + ΔE_int_ + ΔE_GB_ + ΔE_surf_ (1)

where G_comp_, G_pro_ and G_lig_ denotes the free energy of the complex, protein, and the ligand; by splitting the energy contribution, it referred to ΔE_ele_, ΔE_vdw_ and ΔE_int_ as the gas-phase interaction energy between protein and ligand, thus including the electrostatic energy term, the van der Waals energy term, and the bond, angle, and dihedral terms, respectively. On the other hand, ΔE_GB_ and ΔE_surf_ indicate the polar and nonpolar desolvation free energy, respectively. The implicit solvation was calculated using the GB model [52], and the non-polar solvation energy was calculated using the solvent accessible surface area algorithm. The ΔGbind reported in this study omitted the entropy contribution due to its relatively high computational demand and the lack of information of the conformational entropy that could lead to the introduction of additional error into the results [53].

### 3.2. Structure-Based Virtual Screening of [1,2,3]triazolo[4,5-h][1,6]naphthyridines, and [1,2,3]triazolo[4,5-b]Pyridines on gD-Pocket 1 and gD-Pocket 2

The MDs trajectories were clustered based on the RMSD matrix of backbone atoms, and we obtained three representative structures for each complex. After removing the HVEM and Nectin-1 structures, we used a total of six gD conformations for the SBVS. The target binding sites were defined by a regular grid of about 20 Å centered on the residues responsible for binding with the cell receptor [8,40,41,54]. The residues that defined the gD binding site at the interface with HVEM, called for clarity “gD-Pocket 1”, were as follows: Ala7, Ser8, Leu9, Lys10, Met11, Ala12, Asp13, Pro14, Asn15, Val24, Leu25, Asp26, Gln27, Leu28, Thr29, Asp30, Pro31, and Pro32. The gD binding site at the interface with Nectin-1, called “gD-Pocket 2”, was characterized by the following residues: Pro23, Leu25, Gln27, Arg36, Val37, Tyr38, His39, Gln132, Val214, Asp215, Ser216, Ile217, Gly218, Met219, Leu220, Pro221, Arg222, Phe223, Thr230, Val231, and Tyr234. 

The selected ligands were taken from an in-house small library of [1,2,3]triazolo[4,5-*h*][1,6]naphthyridines **1–12** including also their synthetic precursor [1,2,3]triazolo[4,5-*b*]pyridines **13–18** [18]. Moreover, a focused library of 63 triazolo [4,5-*h*][1,6]naphthyridines (**19–81**, Table 1) was also designed, along with the corresponding [1,2,3]triazolo[4,5-*b*]pyridines (**82–85**, Table 1). The 2D structures, reported in Table 1, were drawn using the ChemDraw Ultra 7.0 software and converted into 3D form using the import structures panel from Schrodinger maestro interface.

All compounds were optimized via the Ligprep module, [55] considering their ionization state at pH 7.4, and energy minimized using OPLS_2005 as force field [37].

As reported in other studies [56,57,58,59], the docking simulations of our focused library were computed using the Glide [60] ligand flexible algorithm at the standard-precision (SP) level, generating 10 possible poses for each site. The best docked poses for gD-Pocket 1 and gD-Pocket 2 were submitted to 500 ns of MDs in order to define the structural and energy profile of the best ligands in complex with both gD-Pockets. The simulations were carried out under the above-mentioned conditions. All simulations were performed by Desmond package [44] and “Simulation Interactions Diagram” panel was used as a post-MD analysis tool for exploring protein–ligand interactions. MM/GBSA free energy calculations of the best generated complexes were conducted along 100 frames of 500 ns of MDs. ADME descriptors and pharmacokinetic properties of the promising compounds were predicted by means of SwissADME tool [61].

## 4. Conclusions

In the present computational investigation, the pivotal interactions occurring at the interface of gD and its human surface receptors were carefully explored. Since the availability of X-ray structures of gD in complex with HVEM and Nectin-1, this study aimed at highlighting the key residues involved in the binding interface during MDs. Our reasoning was based on the idea that molecular recognition entails a two-way influence between the interacting partners, whereby the flexibility of gD and its human receptors determined the optimal conformation for the complexes formation. This aspect was better appreciated analyzing the behavior of the complexes after MDs, and the resulting dynamic adaptation was used to define the principal residues responsible for forming a stable complex. MM/GBSA analysis revealed a greater binding affinity of gD towards Nectin-1 with respect to HVEM, given that the rearrangement of N-terminal hairpin is not necessary for gD-Nectin-1 interaction, which instead ensured a more rigid gD-binding pocket. By analyzing HSV-1 PPIs, through the GBPM method, gD N-terminus residues at position 10, 12, 13, 14, 15, 26, 27, 29, 33, and 35 were found pivotal for gD-HVEM binding, whereas nine residues, at position 26, 27, 38, 215, 218, 219, 220, 221, and 222 were relevant for the connection with Nectin-1. Afterwards, an SBVS targeting different conformations of HSV-1 gD binding interfaces was applied. Developing PPI inhibitors is challenging [62], owing to issues such as the general lack of small-molecule starting points for drug design, the typical flatness of the interface, the difficulty of distinguishing real from artefact binding, and the size and character of traditional libraries. In silico approaches are a consolidated strategy to speed up the drug discovery process, as demonstrated during the pandemic emergency, increasing our understanding of how biological systems work and translating this knowledge into new molecules with interesting therapeutic potential. In this context, the inhibition of HSV-1 through targeting viral gD protein represents a good way to act with virus adsorption and membrane fusion. Herein we identified four triazole-pyridines as potential HSV-1 gD inhibitors, with a favourable pharmacokinetic profile and a good theoretical affinity towards all conformations of HSV-1 gD. The surface plasmon resonance technique could provide further information into the mechanism of action at a molecular level and could move forward novel antiviral compounds. Our study suggests a promising basis for the design of a new generation of anti-HSV-1 drugs targeting gD-receptor interfaces.

## Figures and Tables

**Table 1:**
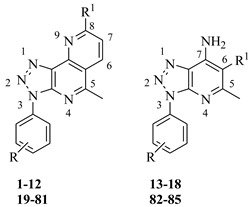
Synthetic (**1–12**) and designed (**19–81**) triazolo [4,5-*h*][1,6]naphthyridines and synthetic (**13–18**) and designed (**82–85**) triazolo [4,5-*b*] pyridines.

Cpd	R	R^1^	Cpd	R	R^1^
**1**	H	Me	**10**	3,4,5-(OMe)_3_	Ph
**2**	H	Ph	**11**	3,4,5-(OMe)_3_	2-OMe-Ph
**3**	H	2-OMe-Ph	**12**	3,4,5-(OMe)_3_	4-OMe-Ph
**4**	H	4-OMe-Ph	**13**	H	COOEt
**5**	4-OMe	Me	**14**	H	CH_2_OH
**6**	4-OMe	Ph	**15**	4-OMe	COOEt
**7**	4-OMe	2-OMe-Ph	**16**	4-OMe	CH_2_OH
**8**	4-OMe	4-OMe-Ph	**17**	3,4,5-(OMe)_3_	COOEt
**9**	3,4,5-(OMe)_3_	Me	**18**	3,4,5-(OMe)_3_	CH_2_OH
					
**19**	H	2-F-Ph	**55**	4-Cl	2-OMe-Ph
**20**	H	2-Br-Ph	**56**	4-Cl	2-F-Ph
**21**	H	2-Cl-Ph	**57**	4-Cl	2-Br-Ph
**22**	H	2-NO_2_-Ph	**58**	4-Cl	2-Cl-Ph
**23**	H	2-NH_2_-Ph	**59**	4-Cl	2-NO_2_-Ph
**24**	H	2-CF_3_-Ph	**60**	4-Cl	2-NH_2_-Ph
**25**	H	2,3-(OMe)_2_-Ph	**61**	4-Cl	2-CF_3_-Ph
**26**	H	2,4-(OMe)_2_-Ph	**62**	4-Cl	2,3-(OMe)_2_-Ph
**27**	H	2,5-(OMe)_2_-Ph	**63**	4-Cl	2,4-(OMe)_2_-Ph
**28**	H	2-OMe-5-Cl-Ph	**64**	4-Cl	2,5-(OMe)_2_-Ph
**29**	H	2-OMe-5-Br-Ph	**65**	4-Cl	2-OMe-5-Cl-Ph
**30**	4-OMe	2-F-Ph	**66**	4-Cl	2-OMe-5-Br-Ph
**31**	4-OMe	2-Br-Ph	**67**	4-F	4-OMe-Ph
**32**	4-OMe	2-Cl-Ph	**68**	4-F	CH_3_
**33**	4-OMe	2-NO_2_-Ph	**69**	4-F	Ph
**34**	4-OMe	2-NH_2_-Ph	**70**	4-F	2-OMe-Ph
**35**	4-OMe	2-CF_3_-Ph	**71**	4-F	2-F-Ph
**36**	4-OMe	2,3-(OMe)_2_-Ph	**72**	4-F	2-Br-Ph
**37**	4-OMe	2,4-(OMe)_2_-Ph	**73**	4-F	2-Cl-Ph
**38**	4-OMe	2,5-(OMe)_2_-Ph	**74**	4-F	2-NO_2_-Ph
**39**	4-OMe	2-OMe-5-Cl-Ph	**75**	4-F	2-NH_2_-Ph
**40**	4-OMe	2-OMe-5-Br-Ph	**76**	4-F	2-CF_3_-Ph
**41**	3,4,5-(OMe)_3_	2-F-Ph	**77**	4-F	2,3-(OMe)_2_-Ph
**42**	3,4,5-(OMe)_3_	2-Br-Ph	**78**	4-F	2,4-(OMe)_2_-Ph
**43**	3,4,5-(OMe)_3_	2-Cl-Ph	**79**	4-F	2,5-(OMe)_2_-Ph
**44**	3,4,5-(OMe)_3_	2-NO_2_-Ph	**80**	4-F	2-OMe-5-Cl-Ph
**45**	3,4,5-(OMe)_3_	2-NH_2_-Ph	**81**	4-F	2-OMe-5-Br-Ph
**46**	3,4,5-(OMe)_3_	2-CF_3_-Ph	**82**	4-Cl	COOEt
**47**	3,4,5-(OMe)_3_	2,3-(OMe)_2_-Ph	**83**	4-Cl	CH_2_OH
**48**	3,4,5-(OMe)_3_	2,4-(OMe)_2_-Ph	**84**	4-F	COOEt
**49**	3,4,5-(OMe)_3_	2,5-(OMe)_2_-Ph	**85**	4-F	CH_2_OH
**50**	3,4,5-(OMe)_3_	2-OMe-5-Cl-Ph			
**51**	3,4,5-(OMe)_3_	2-OMe-5-Br-Ph			
**52**	4-Cl	4-OMe-Ph			
**53**	4-Cl	CH_3_			
**54**	4-Cl	Ph			

**Table 2:** Number of generated clusters, HADDOCK scores, cluster size, Z-score, and BSA values of gD-HVEM and gD-Nectin-1 complexes.

Complex	Cluster	HADDOCK Score	Cluster Size	Z-Score	BSA
gD-HVEM	1	−124.3 ± 5.6	96	−2.1	2144.2 ± 59.6
	2	−89.1± 1.9	24	−0.2	1939.7 ± 69.3
	3	−97.5 ± 7.7	17	−0.7	1925.6 ± 72.1
	4	−93.7 ± 2.1	15	−0.5	1965.3 ± 124.9
	5	−54.1 ± 3.3	10	1.6	1401.2 ± 37.0
	6	−82.2 ± 4.4	9	0.1	1543.9 ± 50.6
	7	−76.3 ± 3.7	7	0.5	1864.7 ± 63.3
	8	−70.1 ± 7.6	6	0.8	1652.0 ± 72.3
	9	−75.7 ± 20.7	4	0.5	1701.5 ± 193.4
gD-Nectin-1	1	−146.0 ± 2.2	178	−1.0	2107.6 ± 20.8
	2	−101.4 ± 3.0	20	1.0	1887 ± 108.6

**Table 3:** Residues involved in the interaction of gD with HVEM and Nectin-1.

	gD-HVEM		gD-Nectin-1	
	gD	HVEM	gD	Nectin-1
Hydrogen bonds	Lys10	Asp7	Asp26	Lys61
	Ala12	Tyr23	Gln27	Lys61
	Pro14	Arg75	Gln27	Thr63
	Asn15	Ser74	Gln132	Gln64
	Gly19	Arg75	Asp215	Asn77
	Gln27	Cys37	Asp215	Asn77
	Thr29	Thr35	Gly218	Gln68
	Lys122	Glu31	Arg222	Gly132
				
Salt bridges	Lys10	Asp7	Arg222	Glu125
	Asp26	Lys26	Asp26	Lys61
	Lys122	Glu31	Arg222	Glu125

**Table 4:** GBPM average scores and quartile distribution of the pivotal hotspots of gD in complex to HVEM and Nectin-1, for all frames of MDs.

gD-HVEM			gD-Nectin-1		
Residue	Average Score	Quartile	Residue	Average Score	Quartile
Ala12	−6.75	Q1	Arg222	−1.80	Q1
Asp13	−2.08	Q1	Asp215	−3.74	Q1
Asn15	−3.13	Q1	Asp26	−1.01	Q1
Asp26	−1.97	Q1	Gln27	−1.13	Q1
Gln27	−5.48	Q1	Gly218	−0.97	Q1
Thr29	−10.00	Q1	Met219	−1.28	Q1
Gly33	−1.21	Q1	Pro221	−1.83	Q1
Arg35	−14.64	Q1	Ser200	−1.85	Q1
Lys10	−0.21	Q2	Tyr38	−1.09	Q1
Lys122	−0.47	Q2	Arg196	−0.20	Q2
Pro14	−0.30	Q2	Arg64	−0.83	Q2
Lys186	−0.20	Q2	Gln132	−0.40	Q2
Gly19	−0.07	Q2	Leu25	−0.44	Q2
Leu257	−0.12	Q2	Lys186	−0.66	Q2
Arg64	−0.20	Q2	Pro23	−0.23	Q2
Ala7	−0.67	Q2	Ser235	−0.64	Q2
Phe17	−0.01	Q3	Tyr137	−0.17	Q2
Lys20	−0.02	Q3	Arg184	−0.04	Q3
Leu25	−0.03	Q3	Asn136	−0.03	Q3
Glu259	−0.02	Q3	Asp139	−0.16	Q3
Asp30	−0.06	Q3	Pro199	−0.03	Q3
Pro32	−0.04	Q3	Ser140	−0.04	Q3
Ala5	−0.04	Q3	Ser216	−0.08	Q3
Glu63	−0.02	Q3	Tyr234	−0.15	Q3
Met11	−0.01	Q4	Val24	−0.05	Q3
Arg18	−0.01	Q4	Ala185	−0.01	Q4
Val24	0.00	Q4	Arg36	−0.01	Q4
Lys245	−0.01	Q4	Asn227	0.00	Q4
Ser258	−0.01	Q4	Ile217	−0.02	Q4
Pro31	0.00	Q4	Lys190	−0.02	Q4
Val34	−0.01	Q4	Phe223	0.00	Q4
Leu4	0.00	Q4	Thr230	−0.01	Q4
			Val231	−0.02	Q4
			Val37	−0.01	Q4

**Table 5:** Predicted ADME parameters: Molecular Weight (MW); Number of H-bond Acceptors and Donors; Octanol/Water Partition Coefficient (logP); Topological Polar Surface Area in A2 (TPSA); and Water Solubility (LogS).

Cpd	MW	H-Bond Acceptors	H-Bond Donors	LogP	TPSA	LogS
**14**	255.28	4	2	1.05	89.85	−2.77
**16**	285.30	5	2	1.06	99.08	−2.82
**83**	289.72	4	2	1.71	89.85	−3.35
**85**	273.27	5	2	1.61	89.85	−2.91

## Data Availability

Additional results found in this work are presented in Supplementary Materials, which will be attached to this document.

## Supplementary Materials

The supporting information can be downloaded at: https://www.mdpi.com/article/10.3390/ijms24087092/s1.
