# Supplementary material for: HSV-1 Glycoprotein D and Its Surface Receptors: Evaluation of Protein–Protein Interaction and Targeting by Triazole-Based Compounds through In Silico Approaches
Source: Int J Mol Sci. 2023 Apr 11;24(8):7092. doi: 10.3390/ijms24087092 (PMC10138673; doi:10.3390/ijms24087092)
Supplement: Supplementary file 1 [file ijms-24-07092-s001.zip › ijms-2291045-supplementary.pdf]

Supporting information

HSV-1 Glycoprotein D and its surface receptors: evaluation of protein-protein interaction and targeting by triazole-based compounds through in silico approaches

Roberta Bivacqua [1], Isabella Romeo [2,3,*], Marilia Barreca [1], Paola Barraja [1], Stefano Alcaro [2,3] and Alessandra Montalbano [1]

[1] Dipartimento di Scienze e Tecnologie Biologiche Chimiche e Farmaceutiche (STEBICEF), Università degli Studi di Palermo, Via Archirafi 32, 90123 Palermo, Italy; roberta.bivacqua@unipa.it (R.B.), marilia.barreca@unipa.it (M.B.), alessandra.montalbano@unipa.it (A.M.), paola.barraja@unipa.it (P.B.).
[2] Dipartimento di Scienze della Salute, Università degli Studi "Magna Græcia" di Catanzaro, Campus "S. Venuta", Viale Europa, 88100 Catanzaro, Italy; isabella.romeo@unicz.it (I.R.), alcaro@unicz.it (S.A.)
[3] Net4Science Academic Spin-Off, Università degli Studi "Magna Græcia" di Catanzaro, Campus "S. Venuta", Viale Europa, 88100 Catanzaro, Italy; isabella.romeo@unicz.it (I.R.), alcaro@unicz.it (S.A.)

*Correspondence: isabella.romeo@unicz.it

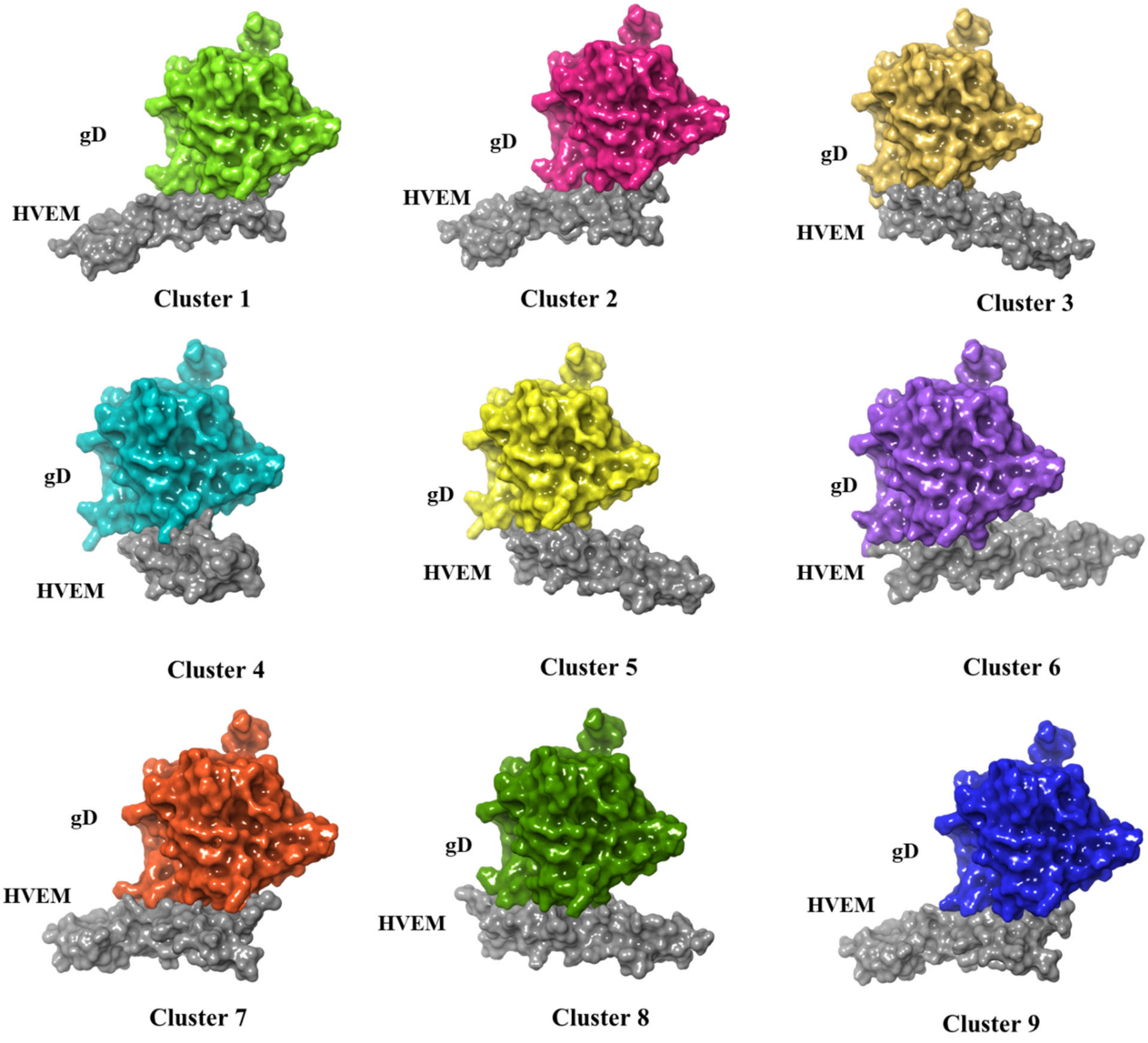

Figure S1. Surface representations of the clusters obtained by HADDOCK of HVEM (gray) in complex with gD (green, pink, faded orange, cyan, yellow, violet, orange, dark green and blue for cluster 1, 2, 3, 4, 5, 6, 7, 8 and 9 respectively).

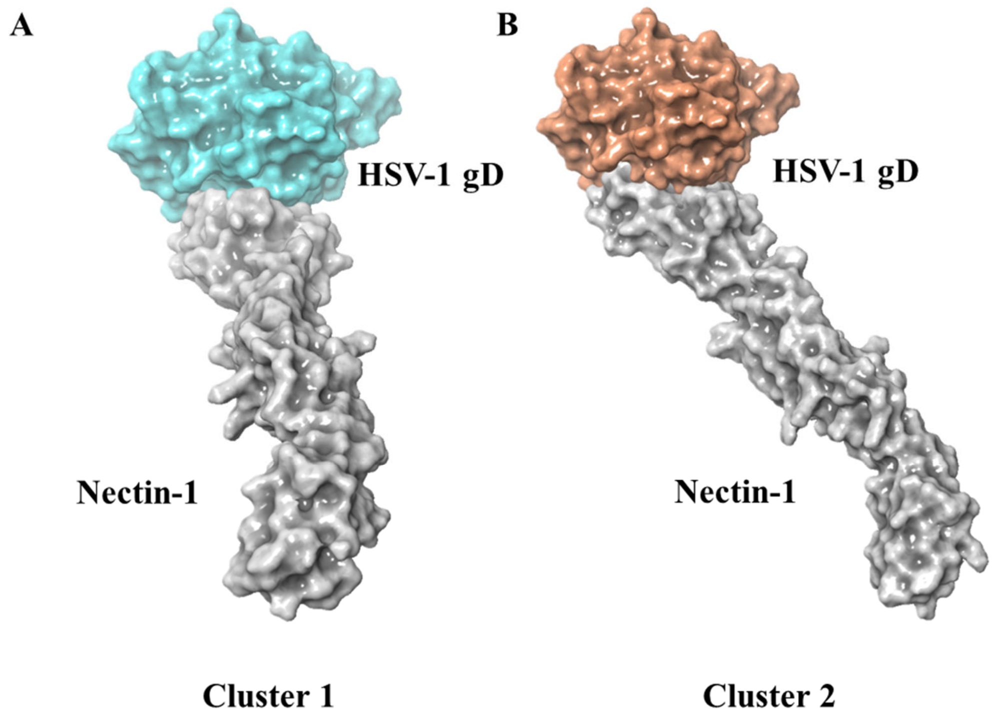

Figure S2. Surface representations of the clusters obtained by HADDOCK of Nectin-1 (gray) in complex with gD (cyan and orange for cluster 1 and 2, respectively).

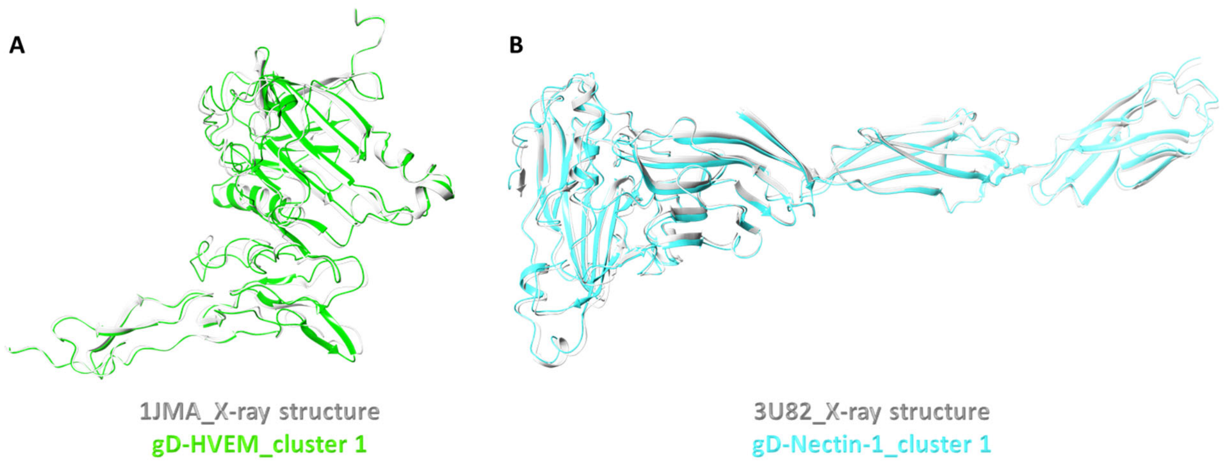

Figure S3. Superpositions between **A**) X-ray structure (PDB code:1JMA) and the obtained best docked pose of gD in complex with HVEM and **B**) X-ray structure (PDB code: 3U82) and the obtained best docked pose of gD in complex with Nectin-1.

Table S1: Matching frames (MF), total GBPM score (TotS), all frames GBPM average scores (AFAveS), and quartile distribution of the pivotal hotspots of gD in complex to HVEM in all frames of MDs.

Residue	#MF	TotS	AFAveS	Quartile
Ala12	922	-6751.66	-6.75	Q1
Asp13	197	-2077.63	-2.08	Q1
Asn15	337	-3132.08	-3.13	Q1
Asp26	264	-1974.60	-1.97	Q1
Gln27	911	-5481.98	-5.48	Q1
Thr29	938	-10004.70	-10.00	Q1
Gly33	607	-1212.89	-1.21	Q1
Arg35	921	-14635.20	-14.64	Q1
Lys10	22	-205.12	-0.21	Q2
Lys122	50	-471.54	-0.47	Q2
Pro14	34	-301.83	-0.30	Q2
Lys186	12	-200.25	-0.20	Q2
Gly19	4	-66.18	-0.07	Q2
Leu257	11	-116.85	-0.12	Q2
Arg64	22	-196.39	-0.20	Q2
Ala7	144	-668.89	-0.67	Q2
Phe17	1	-6.81	-0.01	Q3
Lys20	2	-20.76	-0.02	Q3
Leu25	1	-34.69	-0.03	Q3
Glu259	2	-15.48	-0.02	Q3
Asp30	8	-61.83	-0.06	Q3
Pro32	33	-37.19	-0.04	Q3
Ala5	4	-39.94	-0.04	Q3
Glu63	2	-24.34	-0.02	Q3
Met11	7	-9.03	-0.01	Q4
Arg18	7	-13.35	-0.01	Q4
Val24	1	-0.75	0.00	Q4
Lys245	1	-11.38	-0.01	Q4
Ser258	1	-5.38	-0.01	Q4
Pro31	1	-1.13	0.00	Q4
Val34	1	-6.22	-0.01	Q4
Leu4	3	-3.50	0.00	Q4

Table S2: Matching frames (MF), total GBPM score (TotS), all frames GBPM average scores (AFAveS), and quartile distribution of the pivotal hotspots of gD in complex to Nectin-1 in all frames of MDs.

Residue	#MF	TotS	AFAveS	Quartile
Arg222	629	-3607.74	-1.80	Q1
Asp215	700	-7474.12	-3.74	Q1
Asp26	231	-2012.72	-1.01	Q1
Gln27	271	-2260.99	-1.13	Q1
Gly218	466	-1946.57	-0.97	Q1
Met219	324	-2551.63	-1.28	Q1
Pro221	672	-3651.04	-1.83	Q1
Ser200	344	-3706.21	-1.85	Q1
Tyr38	564	-2184.27	-1.09	Q1
Arg196	37	-393.61	-0.20	Q2
Arg64	149	-1668.43	-0.83	Q2
Gln132	126	-801.80	-0.40	Q2
Leu25	210	-872.21	-0.44	Q2
Lys186	140	-1317.52	-0.66	Q2
Pro23	90	-460.35	-0.23	Q2
Ser235	198	-1272.31	-0.64	Q2
Tyr137	39	-336.19	-0.17	Q2
Arg184	8	-74.78	-0.04	Q3
Asn136	4	-59.69	-0.03	Q3
Asp139	22	-324.78	-0.16	Q3
Pro199	9	-62.42	-0.03	Q3
Ser140	9	-83.69	-0.04	Q3
Ser216	23	-150.97	-0.08	Q3
Tyr234	116	-294.13	-0.15	Q3
Val24	17	-100.52	-0.05	Q3
Ala185	2	-11.58	-0.01	Q4
Arg36	5	-11.36	-0.01	Q4
Asn227	2	-5.78	0.00	Q4
Ile217	6	-35.01	-0.02	Q4
Lys190	7	-38.02	-0.02	Q4
Phe223	2	-3.06	0.00	Q4
Thr230	9	-22.23	-0.01	Q4

Val231	24	-47.55	-0.02	Q4
Val37	12	-23.35	-0.01	Q4

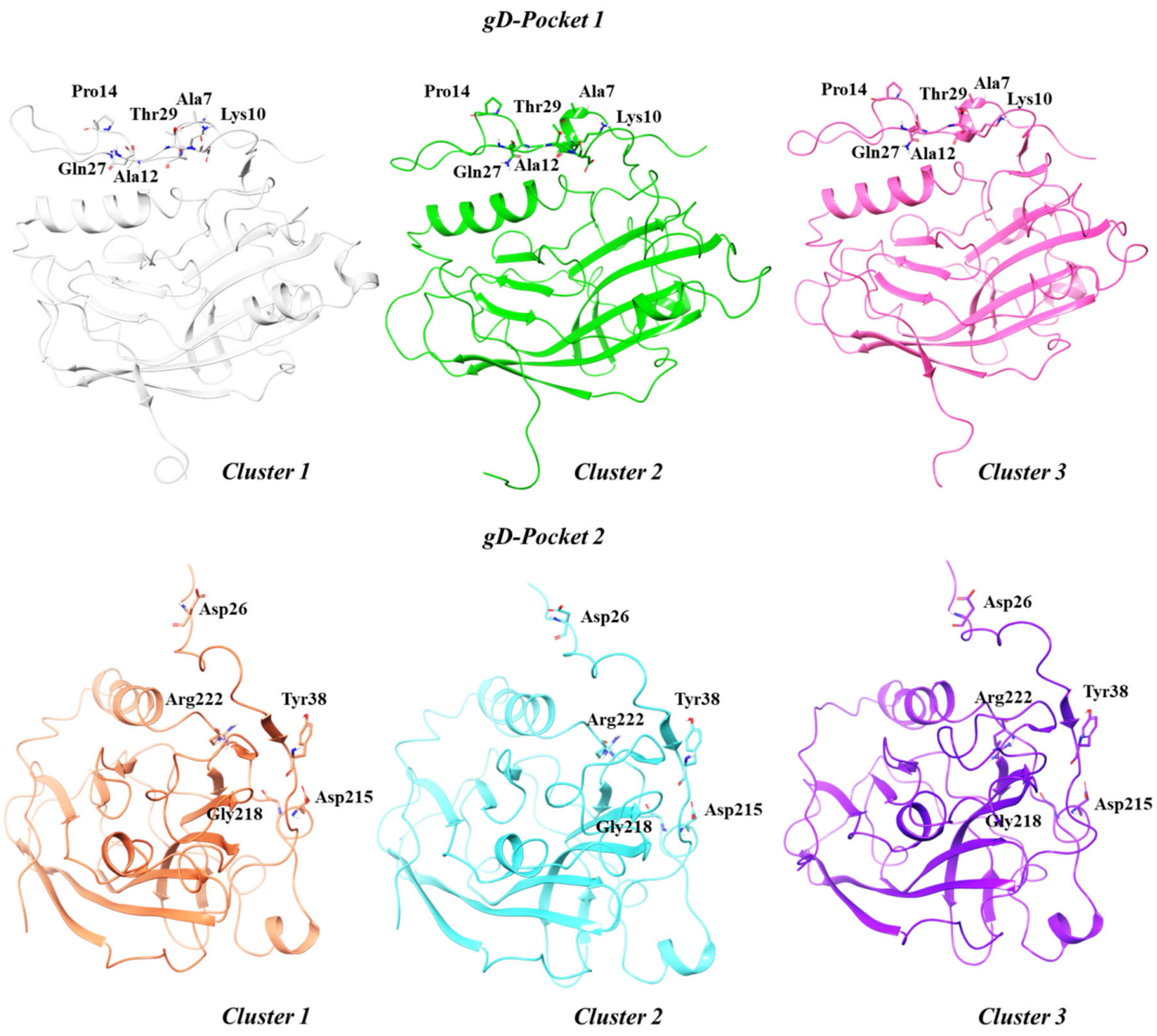

Figure S4. 3D structures of the six representative clusters generated by Desmond Trajectory clustering, after 100 ns of the gD-HVEM (gD-Pocket 1) and gD-Nectin-1 (gD-Pocket 2) MDs. Each structure is represented in cartoon form. The residues involved in crucial contacts with the investigated human receptors, such as HVEM for Pocket 1 and Nectin-1 for Pocket 2, are depicted as carbon sticks.

Table S3. Glide docking score (G-Score) values of 85 compounds against Cluster 1, 2 and 3 for both Pocket 1 and Pocket 2. The compounds are in numerical order. The G-score values are reported in kcal/mol.

	Pocket 1				Pocket 2			
**Cpd**	**Cluster 1**	**Cluster 2**	**Cluster 3**	**Average Gscore value**	**Cluster 1**	**Cluster 2**	**Cluster 3**	**Average Gscore value**
**1**	-4.796	-3.718	-4.174	-4.229	-5.589	-4.899	-4.927	-5.138
**2**	-4.727	-3.806	-3.880	-4.138	-4.740	-4.722	-4.278	-4.580
**3**	-4.999	-3.761	-4.274	-4.345	-4.988	-4.636	-4.569	-4.731
**4**	-4.524	-3.915	-3.773	-4.071	-4.866	-4.959	-4.611	-4.812
**5**	-4.561	-3.435	-3.823	-3.940	-5.255	-4.913	-4.443	-4.870
**6**	-4.544	-3.390	-3.142	-3.692	-4.641	-4.831	-4.038	-4.503
**7**	-4.924	-2.881	-4.160	-3.988	-4.663	-4.385	-4.086	-4.378
**8**	-4.559	-3.052	-2.927	-3.513	-4.748	-4.817	-4.478	-4.681
**9**	-5.007	-3.194	-3.361	-3.854	-4.502	-4.328	-4.132	-4.321
**10**	-4.432	-3.195	-3.063	-3.563	-4.193	-3.404	-3.960	-3.852
**11**	-4.592	-2.958	-3.071	-3.540	-5.059	-4.160	-3.649	-4.289
**12**	-4.415	-2.081	-3.075	-3.190	-4.812	-4.725	-4.344	-4.627
**13**	-6.268	-3.723	-3.919	-4.637	-5.785	-4.882	-4.877	-5.181
**14**	-6.125	-4.623	-5.472	-5.407	-6.281	-5.619	-5.288	-5.729
**15**	-6.302	-3.631	-3.641	-4.525	-5.536	-4.826	-5.029	-5.130
**16**	-6.271	-4.460	-5.460	-5.397	-5.947	-5.105	-5.303	-5.452
**17**	-5.921	-3.082	-3.574	-4.192	-5.168	-5.106	-4.549	-4.941
**18**	-4.802	-4.112	-4.791	-4.568	-5.509	-5.257	-4.727	-5.164
**19**	-4.807	-3.829	-4.033	-4.223	-4.402	-4.453	-4.381	-4.412
**20**	-4.538	-2.943	-3.690	-3.724	-4.618	-4.215	-4.024	-4.286
**21**	-4.726	-3.712	-3.967	-4.135	-4.850	-4.595	-4.245	-4.563
**22**	-5.005	-3.884	-4.349	-4.413	-4.935	-3.467	-4.433	-4.278
**23**	-4.780	-3.405	-4.292	-4.159	-5.320	-4.494	-5.101	-4.972
**24**	-4.807	-3.205	-4.141	-4.051	-4.869	-4.128	-4.355	-4.451
**25**	-4.409	-3.170	-3.894	-3.824	-4.975	-4.622	-4.841	-4.813

**26**	-5.036	-3.389	-3.418	-3.948	-5.018	-4.731	-4.434	-4.728
**27**	-4.670	-3.068	-4.170	-3.969	-4.492	-4.735	-4.085	-4.437
**28**	-4.923	-3.436	-4.386	-4.248	-4.867	-4.248	-4.021	-4.379
**29**	-4.658	-3.266	-4.100	-4.008	-4.578	-4.196	-3.852	-4.209
**30**	-4.672	-3.794	-3.323	-3.930	-4.830	-4.671	-3.962	-4.488
**31**	-4.336	-3.478	-3.133	-3.649	-4.439	-4.407	-3.627	-4.158
**32**	-4.559	-3.705	-3.090	-3.785	-4.679	-4.604	-4.003	-4.429
**33**	-4.827	-3.835	-3.310	-3.991	-4.735	-3.806	-3.962	-4.168
**34**	-4.778	-3.538	-3.706	-4.007	-4.647	-4.641	-4.872	-4.720
**35**	-4.449	-3.115	-3.768	-3.777	-4.762	-4.473	-3.989	-4.408
**36**	-4.756	-2.473	-3.356	-3.528	-4.671	-4.567	-4.182	-4.473
**37**	-4.878	-2.342	-3.027	-3.416	-4.704	-4.688	-4.296	-4.563
**38**	-4.522	-2.678	-3.973	-3.724	-4.602	-4.128	-4.056	-4.262
**39**	-5.005	-2.958	-3.336	-3.766	-4.117	-4.373	-4.040	-4.177
**40**	-4.544	-2.842	-3.322	-3.569	-4.046	-4.079	-3.737	-3.954
**41**	-4.514	-3.130	-2.847	-3.497	-4.885	-3.603	-3.725	-4.071
**42**	-4.171	-2.522	-2.657	-3.117	-3.505	-3.105	-3.220	-3.277
**43**	-4.362	-2.897	-2.833	-3.364	-4.039	-3.830	-3.631	-3.833
**44**	-4.589	-2.828	-3.331	-3.583	-4.029	-3.476	-3.773	-3.759
**45**	-4.545	-3.877	-3.672	-4.031	-4.297	-4.091	-4.379	-4.256
**46**	-4.324	-2.628	-2.951	-3.301	-4.913	-3.316	-3.378	-3.869
**47**	-4.564	-2.521	-3.022	-3.369	-4.004	-3.781	-3.530	-3.772
**48**	-4.602	-2.227	-2.958	-3.262	-4.525	-3.401	-3.424	-3.783
**49**	-4.321	-1.719	-3.446	-3.162	-5.148	-4.228	-3.702	-4.359
**50**	-4.592	-2.726	-3.032	-3.450	-3.918	-3.406	-3.215	-3.513
**51**	-4.503	-2.260	-2.847	-3.203	-3.737	-3.404	-3.665	-3.602
**52**	-4.507	-3.362	-2.714	-3.528	-4.617	-4.867	-4.160	-4.548
**53**	-4.555	-3.982	-3.868	-4.135	-4.870	-5.027	-4.060	-4.652
**54**	-4.573	-3.633	-3.878	-4.028	-4.171	-4.657	-3.585	-4.138
**55**	-4.843	-3.621	-3.949	-4.138	-4.110	-4.035	-4.095	-4.080
**56**	-4.650	-3.560	-4.083	-4.098	-4.155	-3.870	-4.313	-4.113

**57**	-4.393	-3.246	-3.465	-3.701	-4.239	-3.731	-3.950	-3.973
**58**	-4.625	-3.475	-3.817	-3.972	-4.668	-4.676	-4.233	-4.526
**59**	-4.759	-2.992	-3.983	-3.911	-4.292	-3.796	-4.063	-4.050
**60**	-4.688	-3.053	-3.769	-3.837	-4.432	-4.424	-4.778	-4.545
**61**	-4.363	-3.477	-3.934	-3.925	-3.966	-4.056	-3.973	-3.998
**62**	-4.830	-3.340	-3.108	-3.759	-4.300	-4.214	-4.417	-4.310
**63**	-4.890	-3.471	-2.878	-3.746	-4.691	-4.837	-4.065	-4.531
**64**	-4.571	-3.556	-3.994	-4.040	-5.266	-4.068	-4.047	-4.460
**65**	-4.756	-3.454	-4.216	-4.142	-4.180	-3.903	-3.912	-3.998
**66**	-4.463	-3.131	-3.919	-3.838	-4.204	-3.631	-3.605	-3.813
**67**	-4.592	-3.824	-3.040	-3.819	-4.848	-4.876	-4.553	-4.759
**68**	-4.699	-3.480	-3.888	-4.022	-4.869	-4.926	-4.189	-4.661
**69**	-4.766	-3.609	-3.825	-4.067	-4.320	-3.737	-4.360	-4.139
**70**	-4.931	-3.701	-4.017	-4.216	-4.280	-4.470	-4.350	-4.367
**71**	-4.973	-3.767	-4.178	-4.306	-4.049	-4.812	-4.415	-4.425
**72**	-4.608	-3.411	-3.790	-3.936	-4.041	-4.496	-4.036	-4.191
**73**	-4.812	-3.767	-4.115	-4.231	-4.124	-4.750	-4.252	-4.375
**74**	-4.914	-3.817	-4.082	-4.271	-4.372	-3.711	-4.121	-4.068
**75**	-4.718	-3.472	-4.151	-4.114	-5.354	-4.604	-4.844	-4.934
**76**	-4.742	-3.325	-4.014	-4.027	-4.629	-4.550	-4.424	-4.534
**77**	-4.879	-2.742	-2.914	-3.512	-4.779	-4.793	-4.691	-4.754
**78**	-4.932	-3.357	-3.315	-3.868	-4.760	-4.706	-4.083	-4.516
**79**	-4.674	-3.316	-4.078	-4.023	-4.264	-4.710	-4.125	-4.366
**80**	-4.891	-3.387	-4.404	-4.227	-4.136	-4.546	-4.027	-4.236
**81**	-4.642	-3.206	-4.021	-3.956	-3.681	-4.326	-3.742	-3.916
**82**	-6.087	-3.423	-3.668	-4.393	-5.478	-4.877	-4.782	-5.046
**83**	-6.113	-4.422	-5.268	-5.268	-6.070	-5.597	-5.104	-5.590
**84**	-6.308	-3.533	-3.753	-4.531	-5.588	-4.830	-4.773	-5.064
**85**	-6.182	-4.523	-5.407	-5.371	-5.980	-5.121	-5.240	-5.447

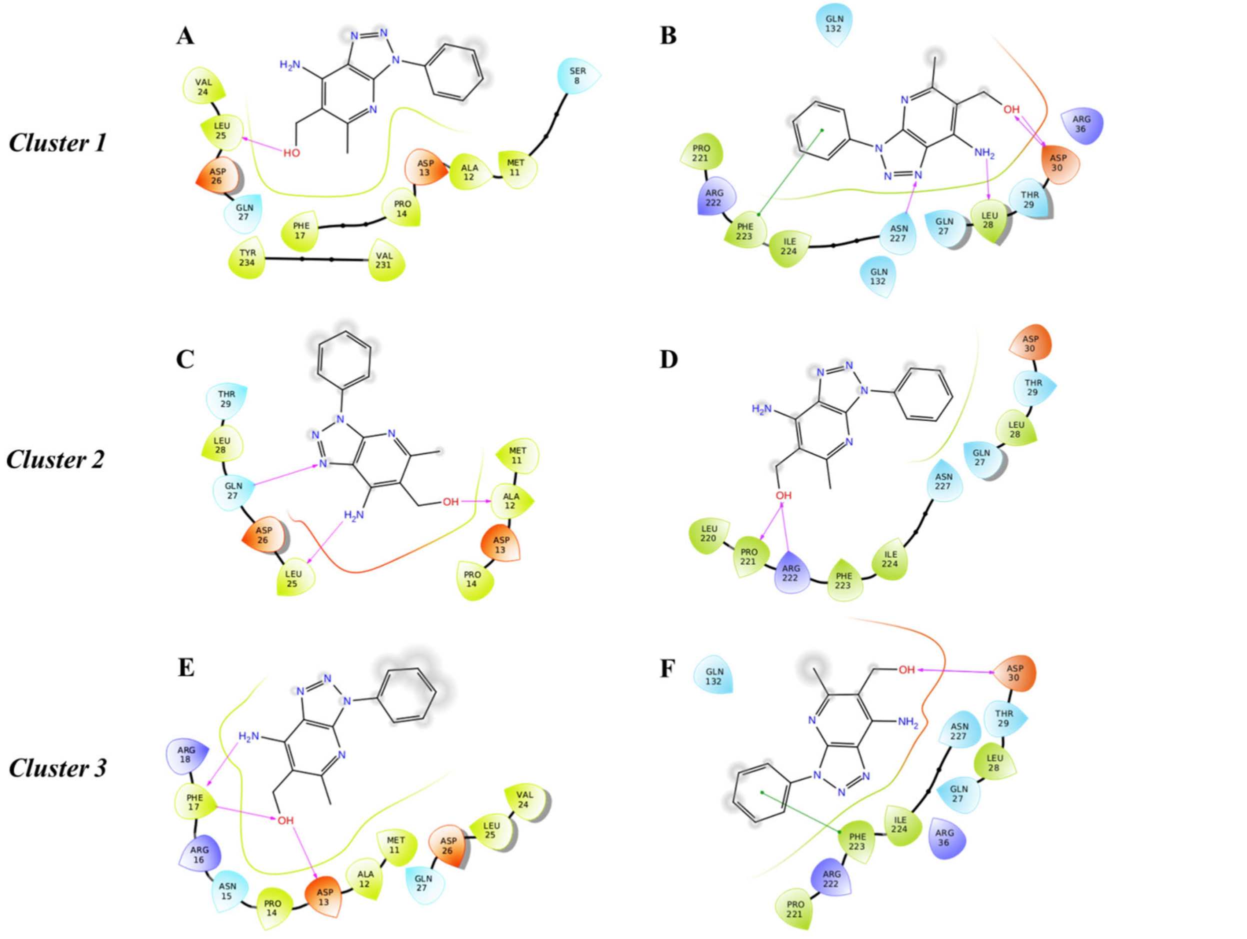

Figure S5. 2D Ligand Interaction diagram of **14** in complex to **A**) cluster 1, **C**) cluster 2, **E**) cluster 3 of gD-Pocket 1, and **14** in complex to **B**) cluster 1, **D**) cluster 2, **F**) cluster 3 of gD-Pocket 2.

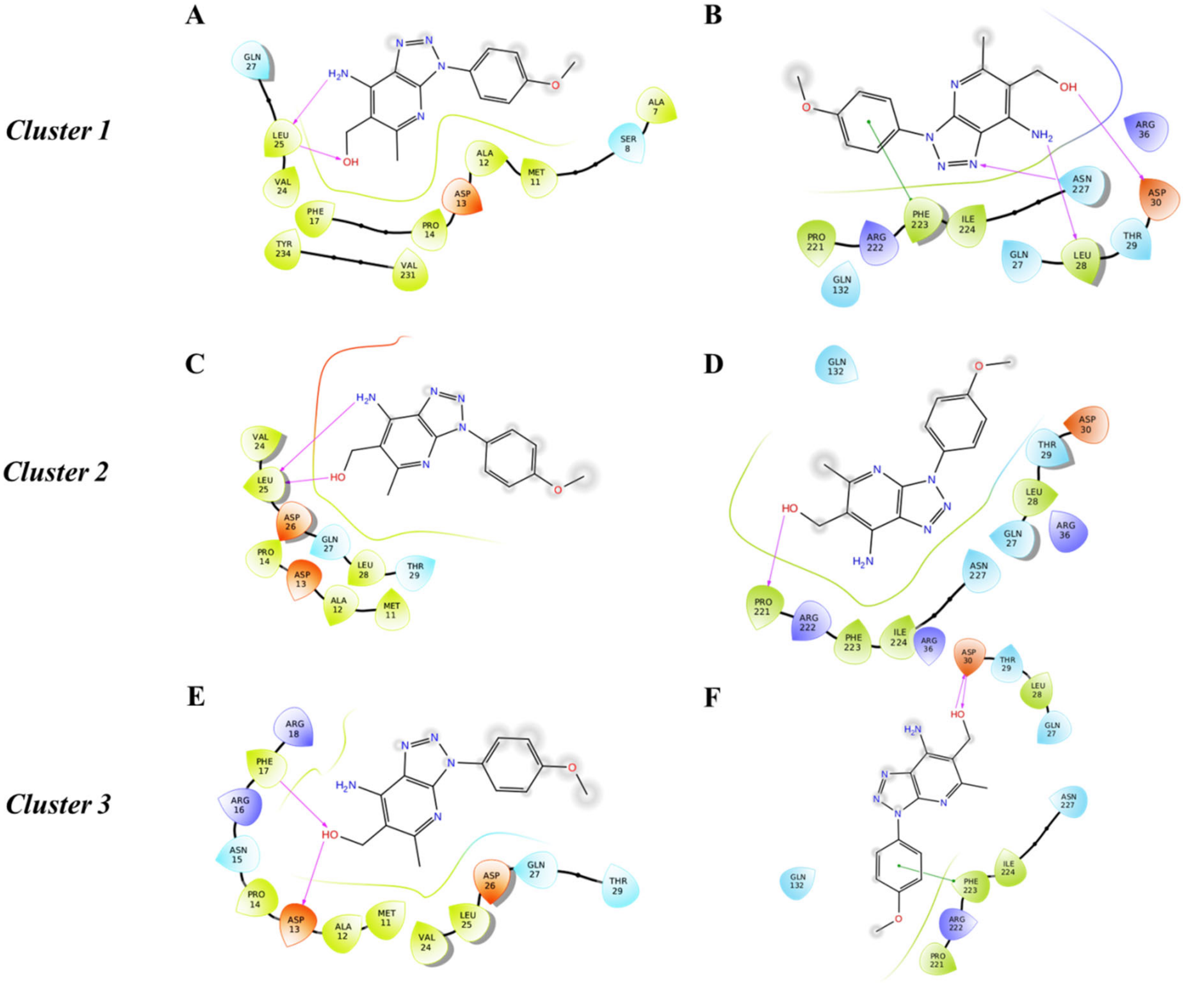

Figure S6. 2D Ligand Interaction diagram of **16** in complex to **A**) cluster 1, **C**) cluster 2, **E**) cluster 3 of gD-Pocket 1, and **16** in complex to **B**) cluster 1, **D**) cluster 2, **F**) cluster 3 of gD-Pocket 2.

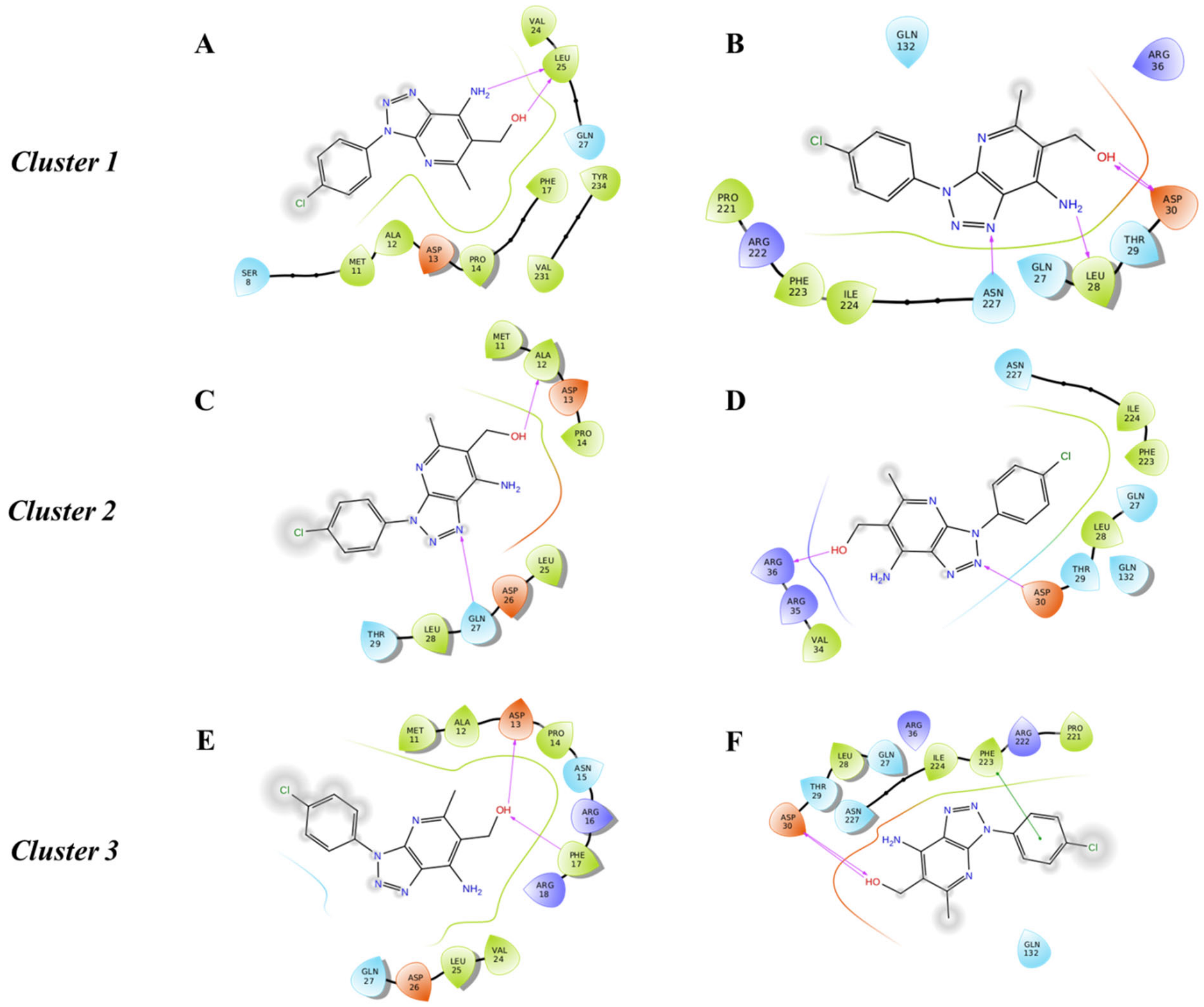

Figure S7. 2D Ligand Interaction diagram of **83** in complex to **A**) cluster 1, **C**) cluster 2, **E**) cluster 3 of gD-Pocket 1, and **83** in complex to **B**) cluster 1, **D**) cluster 2, **F**) cluster 3 of gD-Pocket 2.

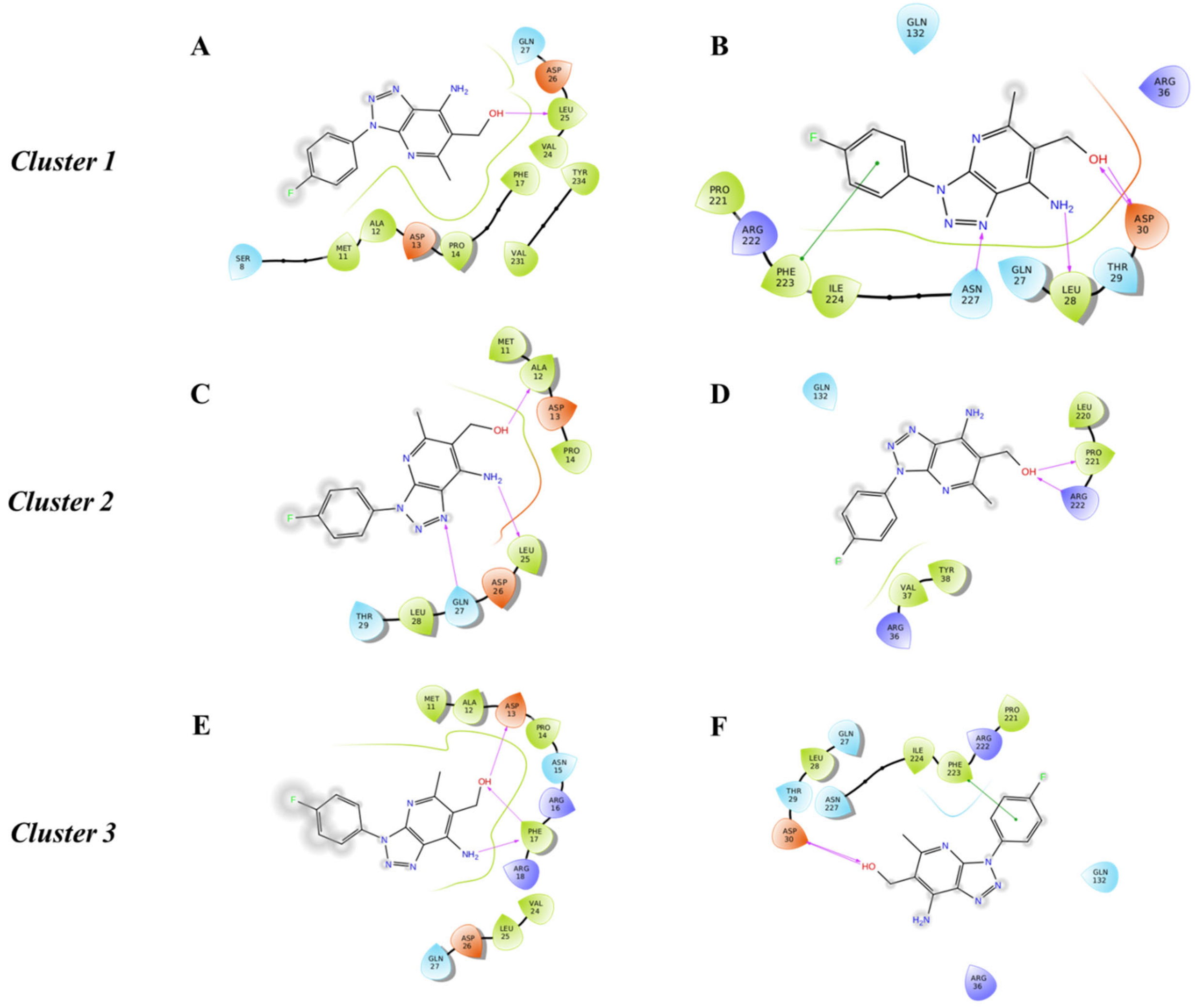

Figure S8. 2D Ligand Interaction diagram of **85** in complex to **A**) cluster 1, **C**) cluster 2, **E**) cluster 3 of gD-Pocket 1, and **85** in complex to **B**) cluster 1, **D**) cluster 2, **F**) cluster 3 of gD-Pocket 2.

SMILES

1	CC(C4=C3N=C(C)C=C4)=NC1=C3N=NN1C2=CC=CC=C2
2	CC(C4=C3N=C(C5=CC=CC=C5)C=C4)=NC1=C3N=NN1C2=CC=CC=C2
3	CC(C4=C3N=C(C5=C(OC)C=CC=C5)C=C4)=NC1=C3N=NN1C2=CC=CC=C2
4	CC(C4=C3N=C(C5=CC=C(OC)C=C5)C=C4)=NC1=C3N=NN1C2=CC=CC=C2
5	CC(C4=C3N=C(C)C=C4)=NC1=C3N=NN1C2=CC=C(OC)C=C2
6	CC(C4=C3N=C(C5=CC=CC=C5)C=C4)=NC1=C3N=NN1C2=CC=C(OC)C=C2
7	CC(C4=C3N=C(C5=C(OC)C=CC=C5)C=C4)=NC1=C3N=NN1C2=CC=C(OC)C=C2
8	CC(C4=C3N=C(C5=CC=C(OC)C=C5)C=C4)=NC1=C3N=NN1C2=CC=C(OC)C=C2
9	CC(C4=C3N=C(C)C=C4)=NC1=C3N=NN1C2=CC(OC)=C(OC)C(OC)=C2
10	CC(C4=C3N=C(C5=CC=CC=C5)C=C4)=NC1=C3N=NN1C2=CC(OC)=C(OC)C(OC)=C2
11	CC(C4=C3N=C(C5=C(OC)C=CC=C5)C=C4)=NC1=C3N=NN1C2=CC(OC)=C(OC)C(OC)=C2
12	CC(C4=C3N=C(C5=CC=C(OC)C=C5)C=C4)=NC1=C3N=NN1C2=CC(OC)=C(OC)C(OC)=C2
13	CC(C(C(OCC)=O)=C3N)=NC1=C3N=NN1C2=CC=CC=C2
14	CC(C(CO)=C3N)=NC1=C3N=NN1C2=CC=CC=C2
15	CC(C(C(OCC)=O)=C3N)=NC1=C3N=NN1C2=CC=C(OC)C=C2
16	CC(C(CO)=C3N)=NC1=C3N=NN1C2=CC=C(OC)C=C2
17	CC(C(C(OCC)=O)=C3N)=NC1=C3N=NN1C2=CC(OC)=C(OC)C(OC)=C2
18	CC(C(CO)=C3N)=NC1=C3N=NN1C2=CC(OC)=C(OC)C(OC)=C2
19	CC(C4=C3N=C(C5=C(F)C=CC=C5)C=C4)=NC1=C3N=NN1C2=CC=CC=C2
20	CC(C4=C3N=C(C5=C(Br)C=CC=C5)C=C4)=NC1=C3N=NN1C2=CC=CC=C2
21	CC(C4=C3N=C(C5=C(Cl)C=CC=C5)C=C4)=NC1=C3N=NN1C2=CC=CC=C2
22	CC(C4=C3N=C(C5=C([N+]([O-])=O)C=CC=C5)C=C4)=NC1=C3N=NN1C2=CC=CC=C2
23	CC(C4=C3N=C(C5=C(N)C=CC=C5)C=C4)=NC1=C3N=NN1C2=CC=CC=C2
24	CC(C4=C3N=C(C5=C(C(F)(F)F)C=CC=C5)C=C4)=NC1=C3N=NN1C2=CC=CC=C2
25	CC(C4=C3N=C(C5=C(OC)C(OC)=CC=C5)C=C4)=NC1=C3N=NN1C2=CC=CC=C2
26	CC(C4=C3N=C(C5=C(OC)C=C(OC)C=C5)C=C4)=NC1=C3N=NN1C2=CC=CC=C2
27	CC(C4=C3N=C(C5=C(OC)C=CC(OC)=C5)C=C4)=NC1=C3N=NN1C2=CC=CC=C2
28	CC(C4=C3N=C(C5=C(OC)C=CC(Cl)=C5)C=C4)=NC1=C3N=NN1C2=CC=CC=C2

29	CC(C4=C3N=C(C5=C(OC)C=CC(Br)=C5)C=C4)=NC1=C3N=NN1C2=CC=CC=C2
30	CC(C4=C3N=C(C5=C(F)C=CC=C5)C=C4)=NC1=C3N=NN1C2=CC=C(OC)C=C2
31	CC(C4=C3N=C(C5=C(Br)C=CC=C5)C=C4)=NC1=C3N=NN1C2=CC=C(OC)C=C2
32	CC(C4=C3N=C(C5=C(Cl)C=CC=C5)C=C4)=NC1=C3N=NN1C2=CC=C(OC)C=C2
33	CC(C4=C3N=C(C5=C([N+]([O-])=O)C=CC=C5)C=C4)=NC1=C3N=NN1C2=CC=C(OC)C=C2
34	CC(C4=C3N=C(C5=C(N)C=CC=C5)C=C4)=NC1=C3N=NN1C2=CC=C(OC)C=C2
35	CC(C4=C3N=C(C5=C(C(F)(F)F)C=CC=C5)C=C4)=NC1=C3N=NN1C2=CC=C(OC)C=C2
36	CC(C4=C3N=C(C5=C(OC)C(OC)=CC=C5)C=C4)=NC1=C3N=NN1C2=CC=C(OC)C=C2
37	CC(C4=C3N=C(C5=C(OC)C=C(OC)C=C5)C=C4)=NC1=C3N=NN1C2=CC=C(OC)C=C2
38	CC(C4=C3N=C(C5=C(OC)C=CC(OC)=C5)C=C4)=NC1=C3N=NN1C2=CC=C(OC)C=C2
39	CC(C4=C3N=C(C5=C(OC)C=CC(Cl)=C5)C=C4)=NC1=C3N=NN1C2=CC=C(OC)C=C2
40	CC(C4=C3N=C(C5=C(OC)C=CC(Br)=C5)C=C4)=NC1=C3N=NN1C2=CC=C(OC)C=C2
41	CC(C4=C3N=C(C5=C(F)C=CC=C5)C=C4)=NC1=C3N=NN1C2=CC(OC)=C(OC)C(OC)=C2
42	CC(C4=C3N=C(C5=C(Br)C=CC=C5)C=C4)=NC1=C3N=NN1C2=CC(OC)=C(OC)C(OC)=C2
43	CC(C4=C3N=C(C5=C(Cl)C=CC=C5)C=C4)=NC1=C3N=NN1C2=CC(OC)=C(OC)C(OC)=C2
44	CC(C4=C3N=C(C5=C([N+]([O-])=O)C=CC=C5)C=C4)=NC1=C3N=NN1C2=CC(OC)=C(OC)C(OC)=C2
45	CC(C4=C3N=C(C5=C(N)C=CC=C5)C=C4)=NC1=C3N=NN1C2=CC(OC)=C(OC)C(OC)=C2
46	CC(C4=C3N=C(C5=C(C(F)(F)F)C=CC=C5)C=C4)=NC1=C3N=NN1C2=CC(OC)=C(OC)C(OC)=C2
47	CC(C4=C3N=C(C5=C(OC)C(OC)=CC=C5)C=C4)=NC1=C3N=NN1C2=CC(OC)=C(OC)C(OC)=C2
48	CC(C4=C3N=C(C5=C(OC)C=C(OC)C=C5)C=C4)=NC1=C3N=NN1C2=CC(OC)=C(OC)C(OC)=C2
49	CC(C4=C3N=C(C5=C(OC)C=CC(OC)=C5)C=C4)=NC1=C3N=NN1C2=CC(OC)=C(OC)C(OC)=C2
50	CC(C4=C3N=C(C5=C(OC)C=CC(Cl)=C5)C=C4)=NC1=C3N=NN1C2=CC(OC)=C(OC)C(OC)=C2
51	CC(C4=C3N=C(C5=C(OC)C=CC(Br)=C5)C=C4)=NC1=C3N=NN1C2=CC(OC)=C(OC)C(OC)=C2
52	CC(C4=C3N=C(C5=CC=C(OC)C=C5)C=C4)=NC1=C3N=NN1C2=CC=C(Cl)C=C2
53	CC(C4=C3N=C(C)C=C4)=NC1=C3N=NN1C2=CC=C(Cl)C=C2
54	CC(C4=C3N=C(C5=CC=CC=C5)C=C4)=NC1=C3N=NN1C2=CC=C(Cl)C=C2
55	CC(C4=C3N=C(C5=C(OC)C=CC=C5)C=C4)=NC1=C3N=NN1C2=CC=C(Cl)C=C2
56	CC(C4=C3N=C(C5=C(F)C=CC=C5)C=C4)=NC1=C3N=NN1C2=CC=C(Cl)C=C2
57	CC(C4=C3N=C(C5=C(Br)C=CC=C5)C=C4)=NC1=C3N=NN1C2=CC=C(Cl)C=C2
58	CC(C4=C3N=C(C5=C(Cl)C=CC=C5)C=C4)=NC1=C3N=NN1C2=CC=C(Cl)C=C2

59	CC(C4=C3N=C(C5=C([N+]([O-])=O)C=CC=C5)C=C4)=NC1=C3N=NN1C2=CC=C(Cl)C=C2
60	CC(C4=C3N=C(C5=C(N)C=CC=C5)C=C4)=NC1=C3N=NN1C2=CC=C(Cl)C=C2
61	CC(C4=C3N=C(C5=C(C(F)(F)F)C=CC=C5)C=C4)=NC1=C3N=NN1C2=CC=C(Cl)C=C2
62	CC(C4=C3N=C(C5=C(OC)C(OC)=CC=C5)C=C4)=NC1=C3N=NN1C2=CC=C(Cl)C=C2
63	CC(C4=C3N=C(C5=C(OC)C=C(OC)C=C5)C=C4)=NC1=C3N=NN1C2=CC=C(Cl)C=C2
64	CC(C4=C3N=C(C5=C(OC)C=CC(OC)=C5)C=C4)=NC1=C3N=NN1C2=CC=C(Cl)C=C2
65	CC(C4=C3N=C(C5=C(OC)C=CC(Cl)=C5)C=C4)=NC1=C3N=NN1C2=CC=C(Cl)C=C2
66	CC(C4=C3N=C(C5=C(OC)C=CC(Br)=C5)C=C4)=NC1=C3N=NN1C2=CC=C(Cl)C=C2
67	CC(C4=C3N=C(C5=CC=C(OC)C=C5)C=C4)=NC1=C3N=NN1C2=CC=C(F)C=C2
68	CC(C4=C3N=C(C)C=C4)=NC1=C3N=NN1C2=CC=C(F)C=C2
69	CC(C4=C3N=C(C5=CC=CC=C5)C=C4)=NC1=C3N=NN1C2=CC=C(F)C=C2
70	CC(C4=C3N=C(C5=C(OC)C=CC=C5)C=C4)=NC1=C3N=NN1C2=CC=C(F)C=C2
71	CC(C4=C3N=C(C5=C(F)C=CC=C5)C=C4)=NC1=C3N=NN1C2=CC=C(F)C=C2
72	CC(C4=C3N=C(C5=C(Br)C=CC=C5)C=C4)=NC1=C3N=NN1C2=CC=C(F)C=C2
73	CC(C4=C3N=C(C5=C(Cl)C=CC=C5)C=C4)=NC1=C3N=NN1C2=CC=C(F)C=C2
74	CC(C4=C3N=C(C5=C([N+]([O-])=O)C=CC=C5)C=C4)=NC1=C3N=NN1C2=CC=C(F)C=C2
75	CC(C4=C3N=C(C5=C(N)C=CC=C5)C=C4)=NC1=C3N=NN1C2=CC=C(F)C=C2
76	CC(C4=C3N=C(C5=C(C(F)(F)F)C=CC=C5)C=C4)=NC1=C3N=NN1C2=CC=C(F)C=C2
77	CC(C4=C3N=C(C5=C(OC)C(OC)=CC=C5)C=C4)=NC1=C3N=NN1C2=CC=C(F)C=C2
78	CC(C4=C3N=C(C5=C(OC)C=C(OC)C=C5)C=C4)=NC1=C3N=NN1C2=CC=C(F)C=C2
79	CC(C4=C3N=C(C5=C(OC)C=CC(OC)=C5)C=C4)=NC1=C3N=NN1C2=CC=C(F)C=C2
80	CC(C4=C3N=C(C5=C(OC)C=CC(Cl)=C5)C=C4)=NC1=C3N=NN1C2=CC=C(F)C=C2
81	CC(C4=C3N=C(C5=C(OC)C=CC(Br)=C5)C=C4)=NC1=C3N=NN1C2=CC=C(F)C=C2
82	CC(C(C(OCC)=O)=C3N)=NC1=C3N=NN1C2=CC=C(Cl)C=C2
83	CC(C(CO)=C3N)=NC1=C3N=NN1C2=CC=C(Cl)C=C2
84	CC(C(C(OCC)=O)=C3N)=NC1=C3N=NN1C2=CC=C(F)C=C2
85	CC(C(CO)=C3N)=NC1=C3N=NN1C2=CC=C(F)C=C2
